# Macrophage Nrf1/NFE2L1-Foxo1 axis controls liver fibrosis by modulation of mitochondrial reprogramming

**DOI:** 10.7150/thno.112337

**Published:** 2026-01-01

**Authors:** Yuanbang Lin, Xiyun Bian, Yao Yao, Jingman Xu, Yingli Cao, Qiong Wu, Wen Ning, Lian Li, Mingwei Sheng, Fengmei Wang

**Affiliations:** 1 Department of General Surgery, Tianjin Medical University General Hospital, Tianjin 300052, China.; 2 Department of Anesthesiology, Tianjin First Central Hospital, Tianjin 300192, China.; 3 Central Laboratory, Tianjin Fifth Central Hospital, Tianjin 300450, China.; 4 Department of Hepatology and Gastroenterology, Tianjin First Central Hospital, Tianjin 300192, China.; 5 School of Public Health, North China University of Science and Technology, Tangshan 063000, China.; 6 College of Life Sciences, State Key Laboratory of Medicinal Chemical Biology, Nankai University, Tianjin 300071, China.

**Keywords:** liver fibrosis, innate immunity, inflammation, mitochondria

## Abstract

**Rationale:** Nuclear factor erythroid 2-like 1 (Nrf1/NFE2L1) is a crucial redox-sensitive factor essential for mitochondrial homeostasis. However, its function in controlling macrophage-associated liver inflammation and fibrosis remains to be fully understood. Herein, this study was conducted to elucidate the roles of macrophage Nrf1 in regulating liver fibrosis.

**Methods:** Expression levels were analyzed in human liver tissues collected from individuals diagnosed with or without liver fibrosis. High-fat diet feeding, carbon tetrachloride injection or bile duct ligation was performed respectively to established three mouse models of liver fibrosis. Myeloid-specific Nrf1-knockout (*Nrf1^M-KO^*) mice were developed to investigate the role and underlying mechanisms of macrophage Nrf1 *in vivo* and *in vitro*.

**Results:** Macrophage Nrf1 expression was markedly reduced in liver samples from both humans and mice with liver fibrosis. The deletion of myeloid Nrf1 remarkably accelerated liver inflammation and fibrosis. Macrophages from *Nrf1^M-KO^* mice exhibited enhanced M1 polarization and mitochondrial dysfunction. Mechanistically, Nrf1 directly binds to Foxo1 and inhibits its transcriptional activity. The target gene *KLF16*, regulated by the Nrf1-Foxo1 complex, is crucial for modulating mitochondrial function and immune response.

**Conclusions:** Our study highlights the functional properties of macrophage Nrf1-Foxo1 axis in controlling mitochondrial reprogramming and liver fibrosis progression.

## Introduction

Liver fibrosis is a generic response triggered by long-term liver damage of various etiologies (cholestasis, viral hepatitis, fatty liver disease, etc.) 1. This process results in cirrhosis featured with fibrous architecture, attendant functional disorder and risk of organ failure 2. Liver cirrhosis is generally recognized as an incurable disease, ranking the 14th most common cause of death worldwide 3. Currently, no effective antifibrotic therapy has been approved other than liver transplantation 4. Thus, expanding knowledge of the precise mechanism of liver fibrosis is under urgent need.

The mechanism of liver fibrosis is a hotly debated issue. It is now becoming clear that uncontrolled immune disorder is the key driving force that accelerates liver fibrosis progression 5. During liver fibrosis development, danger-associated molecular patterns (DAMPs) stimulate both resident macrophages (Kupffer cells) and recruited monocyte-derived macrophages 6. These macrophages, upon activation, secrete a variety of cytokines that contribute to liver parenchymal cell injury, enhance activation of hepatic stellate cells (HSCs), and promote accumulation of extracellular matrix (ECM) 7. As one of the most studied cell types in innate immunity, hepatic macrophages have emerged as central players in the progression and regression of liver fibrosis 8. Activated macrophages exist in two major polarization states, macrophages exhibiting the traditional pro-inflammatory M1 phenotype or the alternative anti-inflammatory M2 phenotype. Upon liver injury, stimuli such as high-fat diet can polarize macrophages exclusively into M1 phenotype characterized by a high expression level of pro-inflammatory cytokines, inciting HSCs to release ECM, resulting in progressive fibrosis 9. Increasing evidence indicates that metabolic shifts between glycolysis and mitochondrial oxidative phosphorylation play a role in macrophage polarization 10-11. M1 macrophages predominantly depend on glycolysis, while M2 macrophages utilize the tricarboxylic acid cycle and oxidative phosphorylation 12. Thus, metabolic reprogramming toward aerobic glycolysis, tricarboxylic acid cycle disruption, and oxidative phosphorylation (OXPHOS) inhibition drives M1 macrophage polarization with pro-inflammatory properties. However, it remains largely unknown how macrophages regulate hepatic metabolic, inflammatory and fibrotic programs.

Vertebrate genomes encode nuclear factor erythroid 2-like factor 1-3 (Nrf1-3). Despite their similar DNA binding mode, Nrf1, Nrf2, and Nrf3 exhibit distinct biological functions and regulate target gene sets that partially overlap. Nrf2, the most famous cap 'n' collar basic region leucine zipper factor, is widely known as the master regulator of antioxidative response in both human and murine inflammatory diseases 13-14. Similar to Nrf2, Nrf1 (also known as NFE2L1) is able to bind to ARE regions and takes center stage in maintaining organ integrity. However, the regulatory mechanisms for Nrf1 activation, target genes, and its requirement for cellular homoeostasis seem to be distinct from Nrf2 15. Nrf1 is mainly located in the endoplasmic reticulum (ER) and can be proteolytically activated by DNA damage inducible 1 homolog 2 (DDI2) under pathological conditions. Activated Nrf1 translocates into the nucleus, leading to the expression increase of essential mitochondrial enzymes, such as mitochondrial transcription factor A (TFAM). Global loss of Nrf1 in mice leads to early embryonic lethality 16. Tissue- or cell type-specific knockout of Nrf1 results in tumor immune escape 17, inflammatory diseases 18 and neurodegenerative diseases including Alzheimer's disease 19. Previous studies identified that silencing macrophage Nrf1 primed macrophages towards M1 polarization by inhibition of signal transducers and activators of transcription 1/3 (STAT1/3) 20. Moreover, Nrf1 inactivation in hepatocytes mediated mitochondrial dysfunction and subsequent alcoholic steatohepatitis development 21. However, the immune- regulatory mechanism specific to macrophage Nrf1 in regulating mitochondrial reprogramming and liver fibrosis remains unknown. Figuring out the precise mechanism of macrophage Nrf1 in liver fibrosis could provide valuable insights, potentially aiding in halting and effectively reversing ongoing liver fibrosis.

Herein, this translational study was designed to gain mechanistic insights into the role of macrophage Nrf1 in the progression of liver fibrosis. Initially, the expression of macrophage Nrf1 was analyzed in multiple mouse models as well as liver fibrosis patient samples. Subsequently, myeloid-specific *Nrf1*-knockout (*Nrf1^M-KO^*) mice were generated to study the contribution of macrophage Nrf1 in three kinds of mouse liver fibrosis models. Third, the underlying mechanisms of Nrf1 in controlling macrophage-associated inflammation were extensively investigated both *in vivo* and *in vitro*.

## Materials and Methods

### Human liver samples

A total of 30 liver samples were collected from hemangioma patients who underwent hepatectomy at the Tianjin Medical University General Hospital (Table S1). 10 patients were diagnosed with liver cirrhosis; 10 patients were diagnosed with metabolic dysfunction-associated steatohepatitis (MASH). The control group included participants with no prior history of diabetes, excessive alcohol intake, or viral hepatitis. Pathologists diagnosed liver fibrosis using H&E and Masson's staining. Liver fibrosis degree was graded by two senior pathologists according to established fibrosis scoring systems. The research framework and procedures for sample collection received approval from the Ethics Committee of Tianjin Medical University General Hospital (Serial number: IRB-2023-KY-072). All patients provided informed consent at recruitment.

### Animal experiments

All mice were housed in a specific pathogen-free (SPF) animal facility, maintained under controlled environmental conditions, including regulated temperature and humidity. They were exposed to a 12 h light-dark cycle and provided unrestricted access to food and water. For high fat diet (HFD)-induced liver fibrosis, 4-week-old male mice were given either a normal chow diet (NCD) consisting of 18.3% protein, 10.2% fat, and 71.5% carbohydrates (Research Diets, New Brunswick, CA) or an HFD containing 18.1% protein, 61.6% fat, and 20.3% carbohydrates (Research Diets) over a period of 26 weeks (Table S2, Figure S1). For liver fibrosis caused by carbon tetrachloride (CCl_4_), male mice between the ages of 6 and 8 weeks were assigned randomly to receive intraperitoneal injections of either 2 ml/kg CCl_4_ (10% v/v in olive oil) or olive oil alone. These injections were administered twice weekly over a period of 8 weeks. Liver fibrosis induced by bile duct ligation (BDL) was studied in 6-8-week-old male mice that underwent surgical procedures. During surgery, the mice were anesthetized with isoflurane, and after disinfecting the skin, a midline incision was made in the abdominal area to access the common bile ducts, which were subsequently tied off using 5-0 sutures. Tissue samples were obtained 4 weeks post-surgery. This research complied with the guidelines outlined in the Guide for the Care and Use of Laboratory Animals published by the National Institutes of Health.

### Construction of myeloid-specific knockout mice

This study utilized male mice with the following genotypes: wild-type (WT), *FloxP-Nrf1* (*Nrf1^FL/FL^*), *FloxP-forkhead box protein o1* (*Foxo1*) (*Foxo1^FL/FL^*), Lyz2-Cre *Nrf1* knockout (*Nrf1^M-KO^*), and Lyz2-Cre *Foxo1* knockout (*Foxo1^M-KO^*), all on a C57BL/6 background acquired from Cyagen. Co. Ltd (Suzhou, China). The floxed alleles were bred to homozygosity to generate the *Nrf1^FL/FL^* and *Foxo1^FL/FL^* mice. *Nrf1^M-KO^* and *Foxo1^M-KO^* were produced by crossing *Nrf1^FL/FL^* and *Foxo1^FL/FL^* with Lyz2-Cre mice (with Cre recombinase expression specifically in myeloid cells), maintaining the C57BL/6 genetic background.

### Primary macrophages isolation, culture and treatment

Recombinant macrophage colony-stimulating factor (M-CSF) (M9170, Sigma-Aldrich) were utilized to differentiate bone marrow-derived macrophages (BMMs) as previously described 21. Femurs and tibias were carefully extracted from sacrificed male mice within a laminar flow hood. Bone marrow cells were subsequently expelled using a 30 G needle attached to a 20 mL syringe filled with Dulbecco's modified eagle's medium (DMEM). Following lysis of red blood cells with Roche buffer solution (11814389001), cells were rinsed with phosphate buffered solution (PBS) and plated in DMEM enriched with M-CSF (20 ng/mL) and fetal bovine serum (FBS) (10%). After four days of culture, cells were rinsed with PBS and refreshed with new medium. By day 7, cells were considered as fully differentiated BMMs. BMMs (1x10^6^) were transfected with CRISPR kruppel-like factor 16 (KLF16) activation, CRISPR TFAM activation, KLF16-siRNA, TFAM-siRNA, and control vector (Santa Cruz Biotechnology) using Lipofectamine^TM^ 3000, following the manufacturer`s instructions (Invitrogen). After 24-48h, the cells were treated with lipopolysaccharide (LPS, 100 ng/mL) for an additional 6h.

### Statistical analyses

A two-tailed Student's t-test was utilized for comparisons between two groups, whereas a one-way analysis of variance (ANOVA) followed by Bonferroni's post hoc test was employed for comparisons among multiple groups. Error bars represent the mean ± standard error of the mean (SEM), with p ≤ 0.05 deemed statistically significant. All statistical analyses were performed utilizing Version 8.0 GraphPad Prism software.

Details on other materials and methods are provided in the Supplementary Materials.

## Results

### The production of Nrf1 is significantly downregulated in macrophages from liver tissues undergoing fibrosis

**Figure 1:**
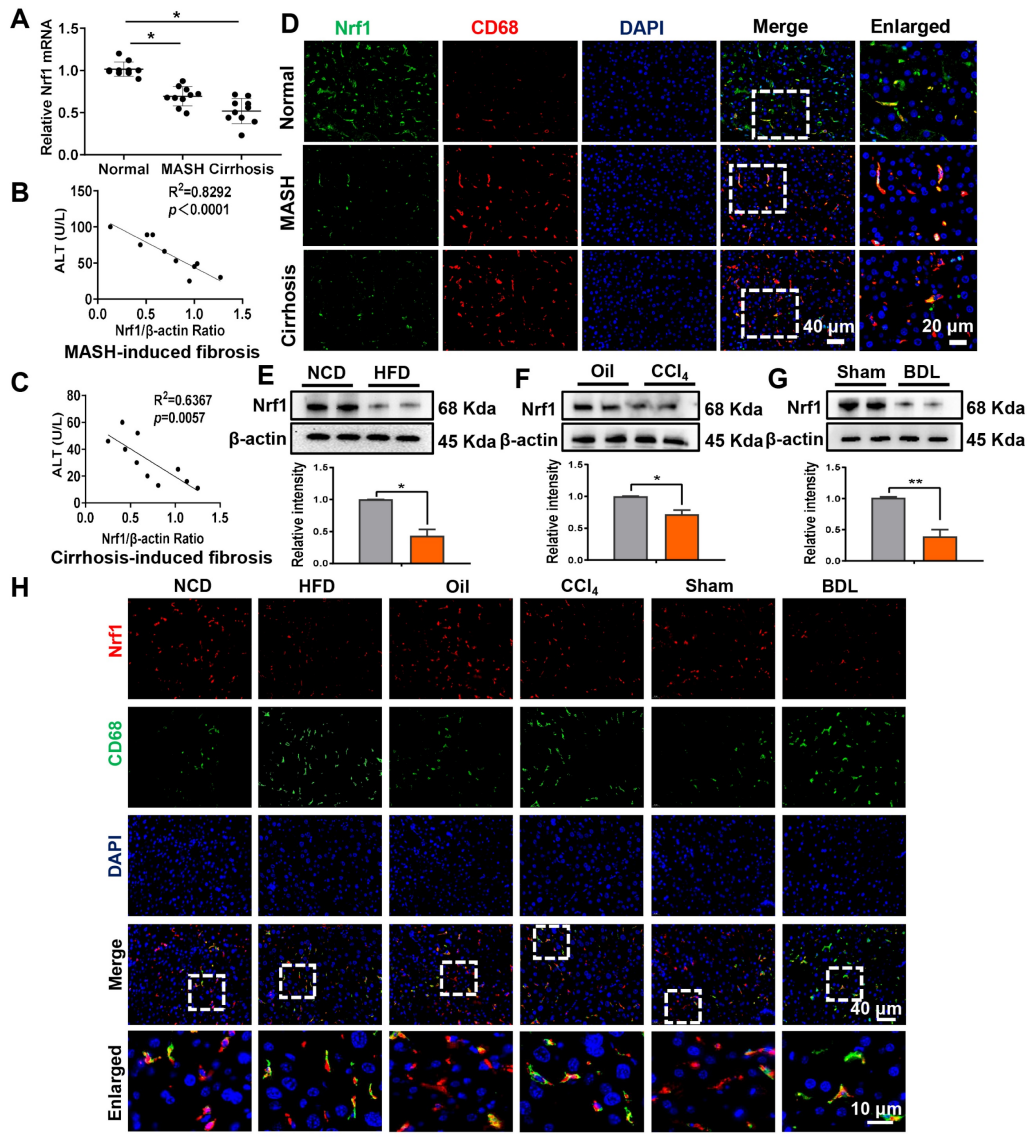
** The expression of Nrf1 is reduced in macrophages within liver fibrotic tissues.** This observation is supported by data obtained from human samples (A-D) and mouse models (E-H): (A) *Nrf1* mRNA levels in liver samples obtained from humans, N = 10/group; The qRT-PCR assay revealed a negative correlation between serum ALT levels and the Nrf1/β-actin ratio in livers from patients with metabolic dysfunction-associated steatohepatitis (MASH) (B) or liver cirrhosis (C), N = 10/group; (D) Dual immunofluorescence analysis of Nrf1 and CD68 in hepatic tissue samples, scale bars: 40 µm, 20 µm, N = 5/group; Nrf1 protein expression was analyzed via WB in three mice livers induced by HFD (E), CCl_4_ injection (F) and BDL (G), N = 6/group; (H) Dual immunofluorescence analysis of Nrf1 and CD68 in hepatic tissue samples, scale bars: 40 µm, 10 µm, N = 5/group. Error bars represent the mean ± standard error of the mean (SEM); *p < 0.05, **p < 0.01, ***p < 0.001.

Initially, our study aimed to analyze Nrf1 expression in fibrotic liver tissues. A total of 30 human liver samples, including normal and fibrotic tissues, were collected for analysis. As indicated by quantitative real-time reverse transcriptase PCR (qRT-PCR) assay, the messenger RNA (mRNA) expression of *Nrf1* was significantly reduced in fibrotic tissues compared to normal tissues (Figure 1A). Interestingly, serum alanine aminotransferase (ALT) values were negatively correlated with Nrf1 expression in liver biopsies from MASH or liver cirrhosis patients, respectively (Figure 1B-C). Using dual immunofluorescence staining, it was observed that Nrf1 expression was largely concentrated in macrophages found in human fibrotic liver tissue (Figure 1D). Then Nrf1 protein levels were assessed in three liver fibrosis models of mice including HFD-induced, CCl_4_-induced and BDL-induced fibrosis. Consistent with the findings in human samples, Nrf1 protein levels were lower in liver samples from all of the mice models than in controls (Figure 1E-G). Fluorescence co-localization of Nrf1 and CD68+ macrophages was detected in three types of liver fibrosis models (Figure 1H). In addition, liver macrophages isolated from fibrotic mouse livers exhibited lower *Nrf1* mRNA levels (Figure S3). Taken together, our data indicated a marked reduction in Nrf1 expression in macrophages within fibrotic liver tissues.

### Myeloid-specific Nrf1 deficiency aggravates liver fibrosis

**Figure 2:**
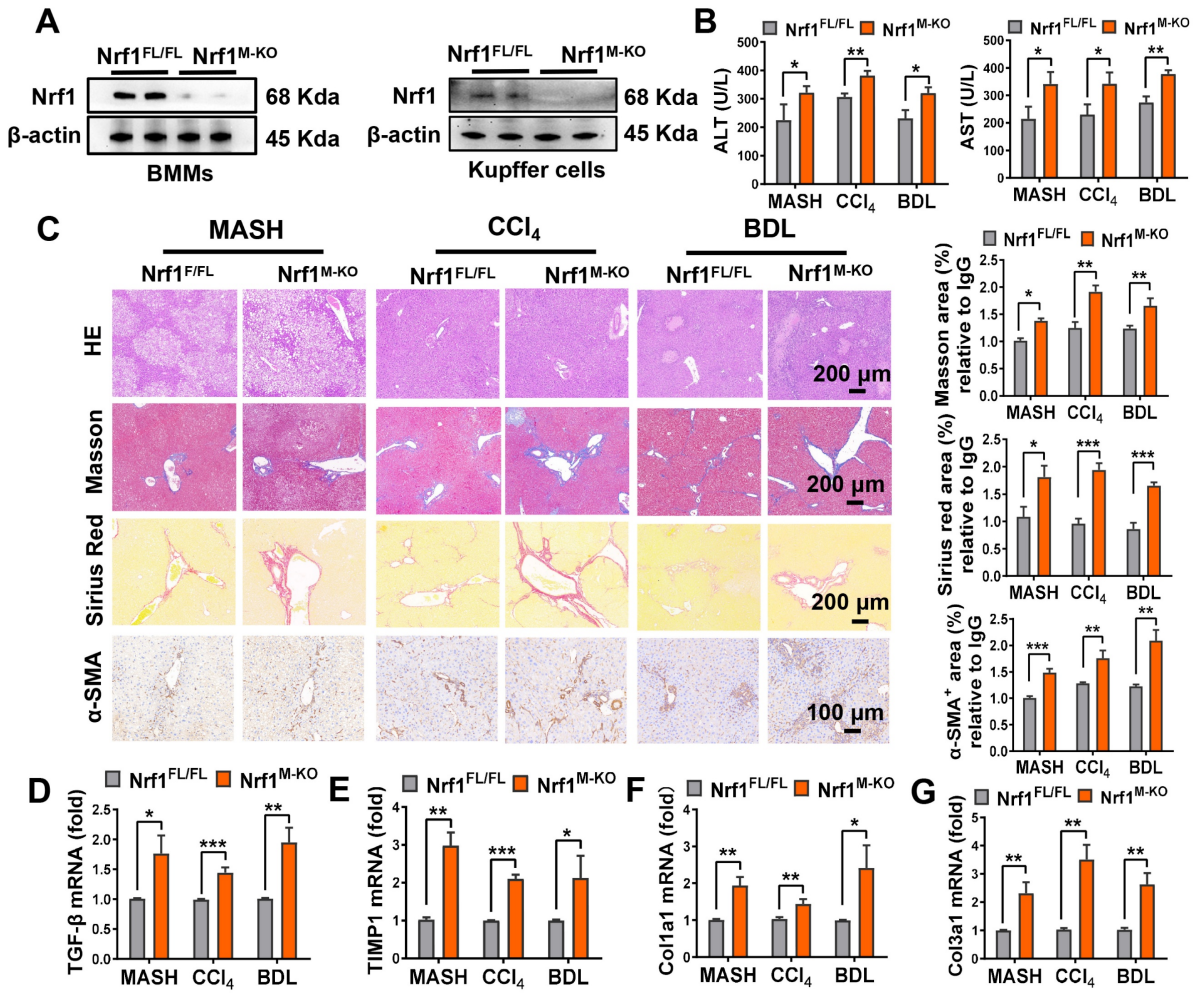
** Myeloid-specific Nrf1 deficiency aggravates mice liver fibrosis.** (A) WB-assisted Nrf1 expression profiles in BMMs and Kupffer cells from *Nrf1^FL/FL^* and *Nrf1^M-KO^* mice, N = 4/group; (B) serum ALT and AST values were measured, N = 6/group; (C) The extent of fibrosis was assessed using H&E, Masson's trichrome, Sirius Red, and α-SMA staining. The positive area of Masson, Sirius Red and α-SMA were quantified, scale bars: 200 µm, 100 µm, N = 5/group; qRT-PCR analysis was conducted to detect mRNA expressions of *TGF-β* (D), *TIMP1* (E), *Col1a1* (F) and *Col3a1* (G) in liver samples, N = 8/group. Error bars represent the mean ± standard error of the mean (SEM); *p < 0.05, **p < 0.01, ***p < 0.001.

To elucidate the role of macrophage-specific Nrf1 in liver fibrosis more precisely, we utilized the Cre-LoxP system to generate mice with myeloid-specific knockout of *Nrf1* (*Nrf1^M-KO^*). As expected, the Nrf1 expression was lacking in both BMMs and Kupffer cells from the *Nrf1^M-KO^* but not the *Nrf1^FL/FL^* mice (Figure 2A). Subsequently, *Nrf1^FL/FL^* and *Nrf1^M-KO^* mice were subjected to HFD feeding, CCl_4_ injection or BDL surgery to trigger liver fibrosis. The *Nrf1^M-KO^* group exhibited elevated serum ALT and aspartate aminotransferase (AST) levels (Figure 2B), indicating exacerbated hepatocellular injury. Moreover, *Nrf1^M-KO^* mice displayed more severe histopathological damage and liver fibrosis than *Nrf1^FL/FL^* mice, as evidenced by H&E, Masson's trichrome, Sirius red, and α-smooth muscle actin (α-SMA) staining (Figure 2C). Specifically, *Nrf1^M-KO^* mice showed significantly higher Ishak scores compared to the controls (Figure S7). In qRT-PCR, mRNA levels of fibrotic factors genes including transforming growth factor-beta1 (TGF-β), collagen type I α1 chain (Col1a1), tissue inhibitor of metalloproteinase 1 (TIMP1), and collagen type III α1 chain (Col3a1) were significantly upregulated in liver tissues of *Nrf1^M-KO^* mice compared to their littermate controls (Figure 2D-G). These findings highlight that the absence of myeloid Nrf1 exacerbates liver fibrosis in mice.

### Myeloid Nrf1 deficiency promotes inflammatory response and mitochondrial impairment in fibrotic livers

**Figure 3:**
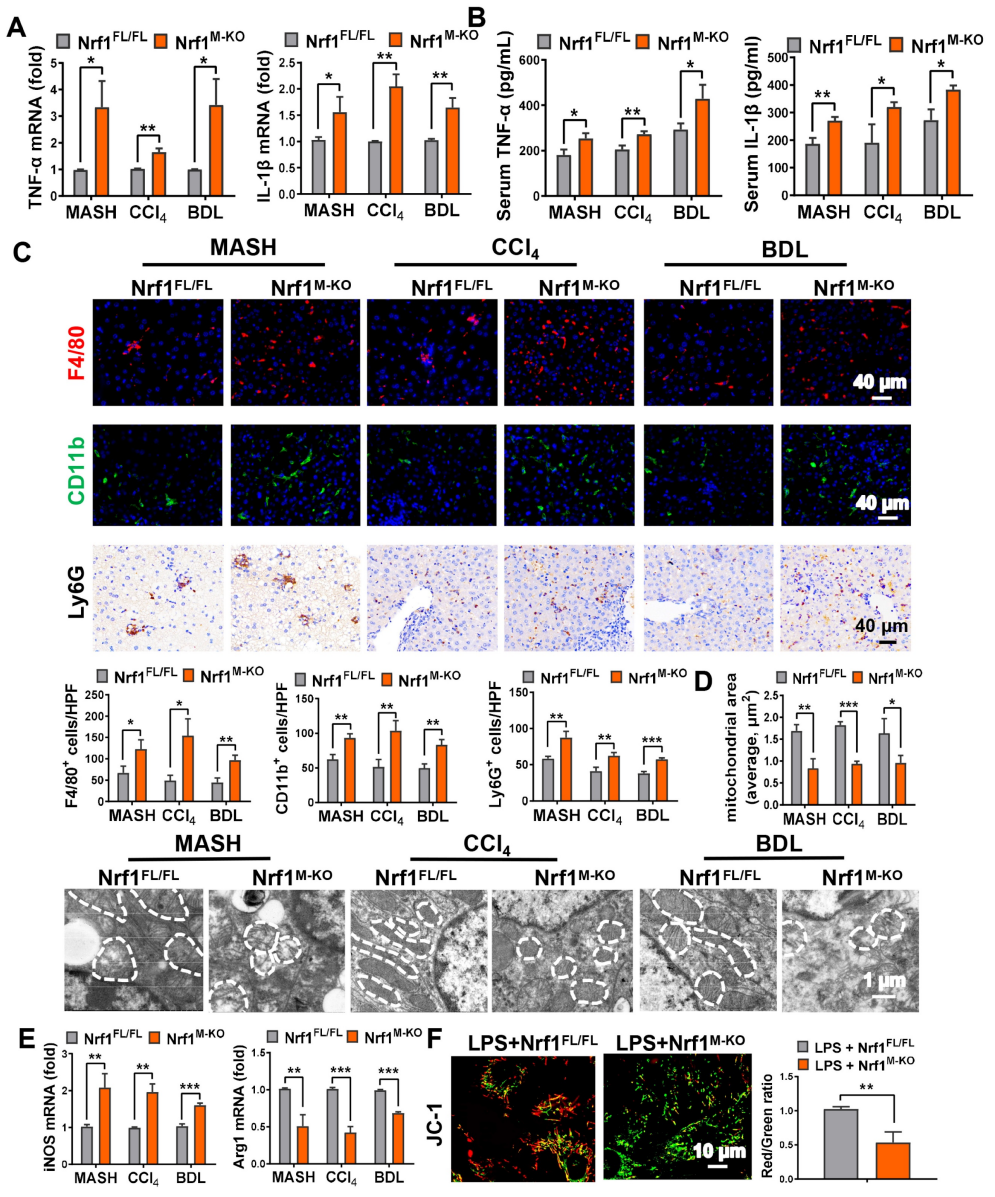
** Myeloid Nrf1 deficiency promotes inflammatory response and mitochondrial impairment in fibrotic livers.** (A) The mRNA levels of *TNF-α* and *IL-1β* were analyzed in liver samples, N = 8/group; (B) serum TNF-α and IL-1β were measured by ELISA, N = 6/group; (C) Immunofluorescence was performed to detect F4/80, CD11b, and Ly6G, and the positive cells were quantified as counts per high power field (HPF), scale bars: 40 µm, N = 5/group; (D) Representative TEM images of liver mitochondria and the quantification of mitochondrial area, scale bar: 1 µm, N = 6/group; (E) Real-time PCR analysis of mRNA expressions of* iNOS* and *Arg1* in liver samples, N = 8/group; Bone marrow-derived macrophages (BMMs) obtained from *Nrf1^FL/FL^* or *Nrf1^M-KO^* mice were incubated with LPS for 6 h. (F) The aggregation/monomer (Red/Green) ratio stained with JC-1; (G) mtDNA released outside from mitochondria; (H) Oxygen consumption rate (OCR) in BMMs, N = 6/group; Error bars represent the mean ± standard error of the mean (SEM); *p < 0.05, **p < 0.01, ***p < 0.001.

Subsequently, we examined how the absence of Nrf1 in macrophages influences inflammatory responses within fibrotic liver tissues. As shown in Figure 3A, the levels of *tumor necrosis factor-α (TNF-α)* and *interleukin-1β (IL-1β)* were significantly elevated in liver samples obtained from *Nrf1^M-KO^* mice compared to control groups. Similarly, Figure 3B illustrates that serum concentrations of TNF-α and IL-1β were markedly higher in *Nrf1^M-KO^* mice relative to their littermate counterparts. To assess the infiltration of inflammatory cells in fibrotic liver tissues, we analyzed macrophage markers F4/80 and CD11b, alongside the neutrophil marker Ly6G. In comparison with *Nrf1^FL/FL^* mice, myeloid-specific deletion of Nrf1 resulted in a pronounced rise in macrophage and neutrophil accumulation within fibrotic livers (Figure 3C). Transmission electron microscopy (TEM) analysis revealed that mitochondrial lesions including mitochondrial cristae disruption, swollen mitochondria and outer membrane rupture were observed in fibrotic liver tissues. The degree of mitochondrial damage was aggravated in Nrf1^M-KO^ fibrotic livers (Figure 3D). In the *Nrf1^M-KO^* mice, mRNA expression level of M1 macrophage marker inducible nitric oxide synthase (iNOS) was upregulated, whereas that of M2 macrophages marker arginase-1 (Arg1) was downregulated in the fibrotic livers, suggesting that Nrf1 deficiency may promote M1 polarization (Figure 3E). In addition, a more detailed examination of the mitochondrial phenotype was conducted in BMMs. Consistent with *in vivo* data, myeloid Nrf1 deficiency exhibited severe mitochondrial dysfunction, as evidenced by decreased JC-1 ratio compared to the controls (Figure 3F).

### Disruption of myeloid Foxo1 suppresses M1 polarization and mitochondrial dysfunction in fibrotic livers

**Figure 4:**
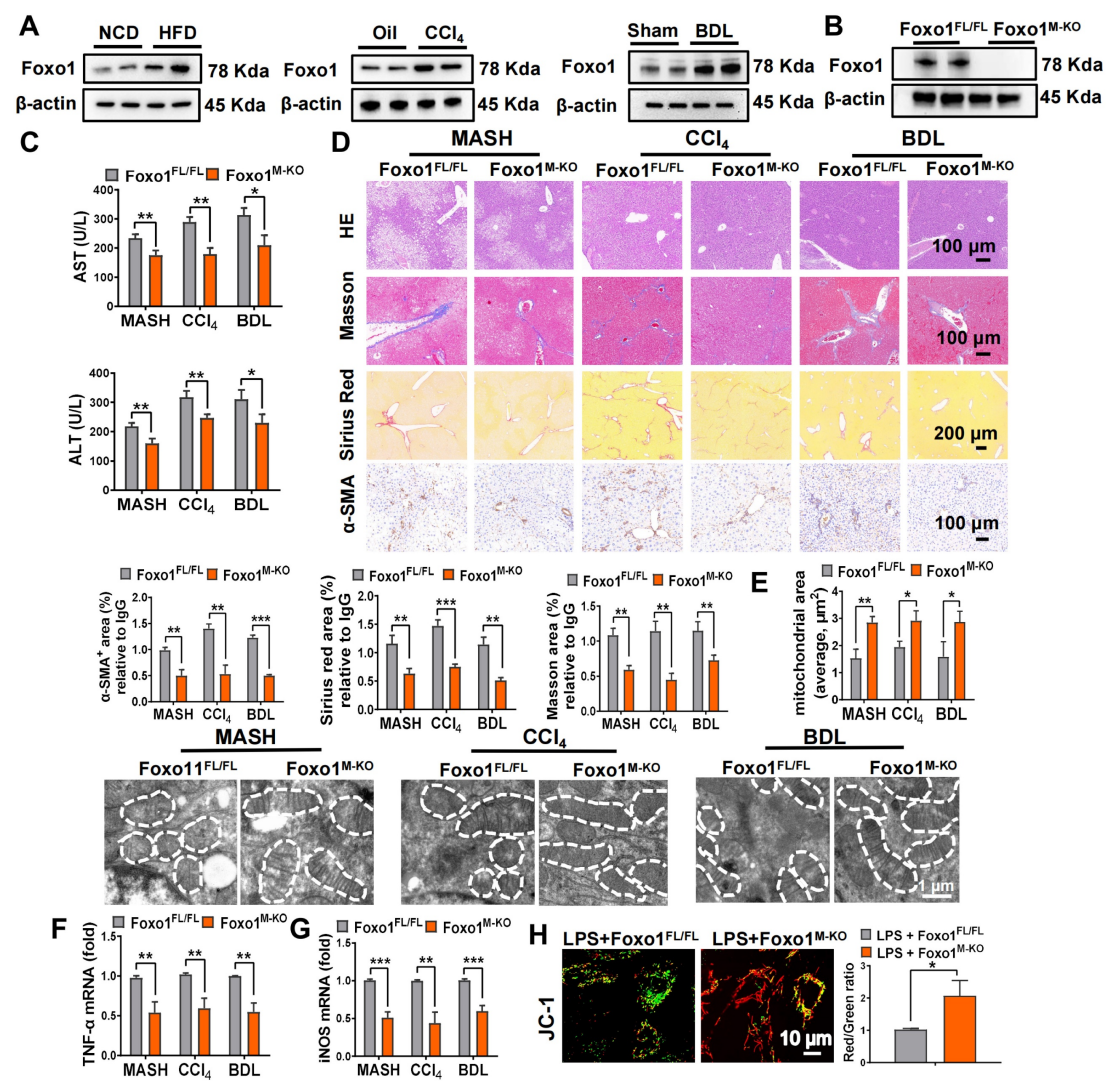
** Disruption of Myeloid Foxo1 suppresses M1 polarization and mitochondrial dysfunction in fibrotic livers.** (A) Protein expression of Foxo1 in mice livers induced by HFD, CCl_4_ injection and BDL, N = 6/group; (B) WB-assisted Foxo1 expression profiles in BMMs from Foxo1^FL/FL^ and Foxo1^M-KO^ mice, N = 4/group; (C) serum ALT and AST values were measured, N = 6/group; (D) Fibrosis severity was evaluated through H&E, Masson's trichrome, Sirius Red, and α-SMA staining methods. Masson positive area, Sirius Red positive area and α-SMA positive area was quantified representively, scale bars: 200 µm, 100 µm, N = 5/group; (E) Representative TEM images of liver mitochondria and the quantification of mitochondrial area, scale bar: 1µm, N = 6/group; Real-time PCR analysis was conducted to detect mRNA expressions of TNF-α (F) and iNOS (G) in liver samples, N = 8/group; Bone marrow-derived macrophages (BMMs) from *Foxo1^FL/FL^* or *Foxo1^M-KO^* mice were cultured with LPS establish in vitro model. (H) The aggregation/monomer (Red/Green) ratio stained with JC-1; (I) mtDNA released outside from mitochondria; (J) oxygen consumption rate (OCR) in BMMs, N = 6/group; Error bars depict mean ± standard error of the mean (SEM), *p < 0.05, **p < 0.01, ***p < 0.001.

As Foxo1 signaling can be activated under acute/chronic liver inflammation, we subsequently explored whether Foxo1 influenced inflammatory response and mitochondrial function in liver fibrosis. Initially, we sought to characterize Foxo1 expression in fibrotic liver samples. Consistent with expectations, fibrotic livers exhibited significantly elevated Foxo1 protein levels compared to normal control livers (Figure 4A). Unlike *Foxo1^FL/FL^* mice, *Foxo1^M-KO^* mice exhibited no Foxo1 expression in BMMs (Figure 4B) and in Kupffer cells reported in our previous work 22-23. The serum levels of ALT and AST (Figure 4C), liver histological lesions, fibrosis severity (Figure 4D, Figure S7) as well as mitochondrial fragmentation (Figure 4E) were inhibited in *Foxo1^M-KO^* mice. Moreover, the mRNA expression of *TNF-α* and *iNOS* was significantly decreased in the *Foxo1^M-KO^* livers, compared to the *Foxo1^FL/FL^* controls (Figure 4F-G), suggesting an improvement of inflammatory response in fibrotic livers. Moreover, Foxo1 deficient BMMs exhibited restored mitochondrial function, as evidenced by increased JC-1 ratio compared to the controls (Figure 4H). These data reveal that myeloid Foxo1 deficiency suppresses M1 polarization and mitochondrial dysfunction in fibrotic livers.

### Nrf1 interacts directly with Foxo1 and modulates KLF16 transcription in macrophages

**Figure 5:**
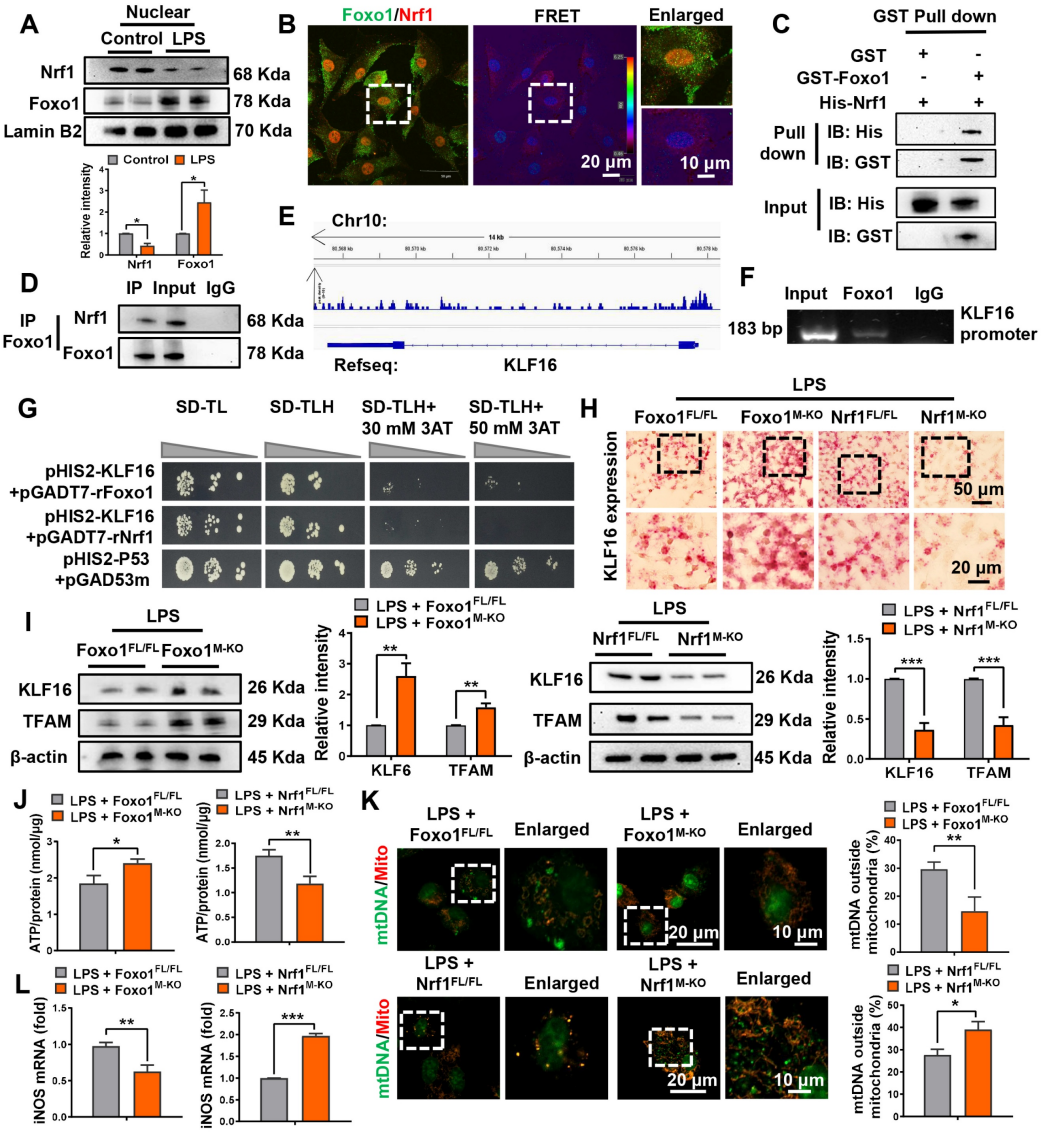
** Nrf1 directly binds with Foxo1 and regulates the transcription of KLF16 in macrophages.** Bone marrow-derived macrophages (BMMs) isolated from *Nrf1^FL/FL^* or *Nrf1^M-KO^* mice were exposed to LPS for 6 h. (A) Analysis of Nrf1 and Foxo1 in nuclear extracts was conducted using Western blot, N = 6/group; (B) IF staining and fluorescence resonance energy transfer (FRET) analysis of Nrf1 and Foxo1 in macrophages stimulated with LPS. DAPI was applied to stain the nuclei (blue). Scale bars: 20 μm, 10 μm, N = 4/group; (C) Glutathione-S- transferase (GST) Pull-down assay to confirm the interaction between Nrf1 and Foxo1 proteins. (D) Analysis of Nrf1 and Foxo1 interaction in LPS-stimulated macrophages from WT mice was performed using immunoprecipitation; ChIP-sequencing assay (E) and ChIP analysis (F) performed in LPS-treated BMMs using an anti-Foxo1 antibody; (G) The interaction between potential transcription factors and *KLF16* promoter examined via yeast-one-hybrid assay; (H) RNA-ISH was performed to detect *KLF16* mRNA in macrophages using a targeted probe, N = 4/group; (I) Western blotting and relative intensity of KLF16, TFAM, N = 6/group; Measurement of ATP level in BMMs (J); The colocalization analysis of cytosol mtDNA (Picogreen) released from mitochondria (MitoTracker Red) in BMMs, Scale bars: 20 μm, 10 μm, N = 4/group (K); Detection of iNOS (L) by qRT-PCR in macrophages, N = 6/group. Error bars depict mean ± standard error of the mean (SEM), *p < 0.05, **p < 0.01, ***p < 0.001.

To further explore the mechanisms by which macrophage Nrf1 and Foxo1 regulate liver fibrosis, nuclear protein was extracted to explore their distribution in macrophages stimulated by LPS. The results showed that LPS could induce the decrease of Nrf1 but upregulation of Foxo1 in the nucleus (Figure 5A). Double immunofluorescence and fluorescence resonance energy transfer (FRET) analysis revealed that Nrf1 co-localized with Foxo1 in the nucleus (Figure 5B, Figure S8). Glutathione-S-transferase (GST) pull-down experiments clearly confirmed that Nrf1 directly binds to Foxo1 (Figure 5C). Furthermore, immunoprecipitation assay also proved that Nrf1 bound to Foxo1 in macrophages (Figure 5D). Then ChIP coupled to massively parallel sequencing (ChIP-Seq) was performed to explore the potential role of the Nrf1-Foxo1 interaction. Clearly, the genomic loci that Foxo1 binds to were in diverse pathways, culminating in response to oxidative stress, protein localization to nucleus, regulation of mRNA metabolic process and macroautophagy pathway (Figure S5). And *KLF16* gene was ranked in the Top 50 target genes of Foxo1 (Figure 5E, Figure S5). To confirm the ChIP-seq peak in the *KLF16* promoter region, ChIP-PCR was conducted using Foxo1 antibodies in LPS-stimulated BMMs. Following ChIP with Foxo1 antibody, primers were specifically designed to identify the Foxo1 DNA-binding site in *KLF16* promoter through PCR analysis (Figure 5F).

In line with the ChIP results, yeast one-hybrid assay demonstrated that Foxo1 directly binds to the *KLF16* promoter region, facilitating the inhibition of *KLF16* transcription. However, Nrf1 could not bind to KLF16 promoter region (Figure 5G). RNA in situ hybridization assay proved that Foxo1 deficiency enhanced the expression of the *KLF16* transcript in LPS-stimulated macrophages. In contrast, the disruption of Nrf1 diminished *KLF16* transcripts (Figure 5H). Consistently, Foxo1 deficiency promoted protein expressions of macrophage KLF16, TFAM and Manganese superoxide dismutase (SOD2), accompanied by increased ATP production and less mtDNA leakage from mitochondria. The iNOS and cytochrome C (CytC) expression were decreased in Foxo1-deficient macrophages compared with controls (Figure 5I-L, Figure S6). Unlike in *Nrf1^FL/FL^* macrophages, *Nrf1^M-KO^* reduced protein levels of KLF16, TFAM and SOD2, but aggravated mitochondrial injury and macrophage M1 polarization (Figure 5I-L, Figure S6). Collectively, these results indicate that macrophage Nrf1-Foxo1 interaction regulates its target gene *KLF16*, which may be involved in energy imbalance and subsequent liver fibrosis.

### Nrf1-Foxo1 axis-regulated mitochondrial function and M1 polarization requires KLF16

**Figure 6:**
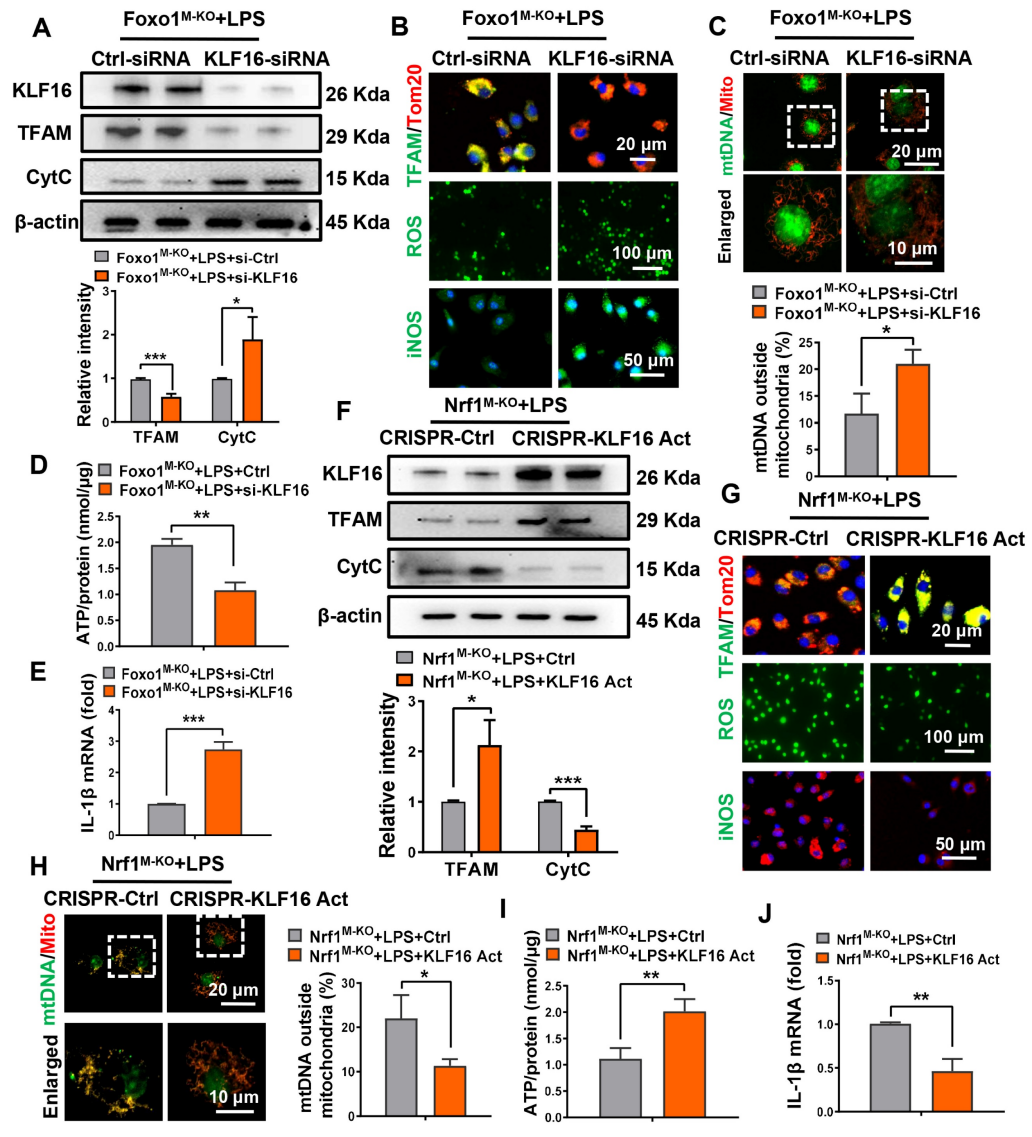
** Nrf1-Foxo1 axis-regulated mitochondrial function and M1 polarization requires KLF16.** Bone marrow-derived macrophages (BMMs) from *Foxo1^M-KO^* or *Nrf1^M-KO^* mice were transfected with KLF16-siRNA or KLF16-activation plasmids following transfection with LPS for 6 h. (A, F) Western-assisted analysis of KLF16, TFAM and CytC, N = 6/group; (B, G) Fluorescence staining of TFAM (green) and Tom20 (red) co-localization, intracellular ROS level (DCFH-DA fluorescence) and iNOS in LPS-stimulated BMMs. Nuclei were visualized in blue using DAPI staining. Scale bars: 20 μm, 100 μm, 50μm, N = 4/group; The colocalization analysis of cytosol mtDNA (Picogreen) released from mitochondria (MitoTracker Red) in BMMs, Scale bars: 20 μm, 10 μm, N = 4/group (C, H); Measurement of ATP level (D, I) in BMMs; (E, J) Detection of IL-1β by qRT-PCR in macrophages, N = 6/group. Error bars depict mean ± standard error of the mean (SEM), *p < 0.05, **p < 0.01, ***p < 0.001.

To further determine the effect of KLF16 on Nrf1-Foxo1 axis-regulated mitochondrial function and macrophage M1 polarization, KLF16-knockdown BMMs were generated, and the reduction of KLF16 expression was confirmed by western blotting. Loss of KLF16 led to the decrease of TFAM and the elevation of CytC (Figure 6A). The abundance of TFAM in mitochondria (marker Tom20) was also reduced by KLF16-siRNA (Figure 6B). Furthermore, the loss of KLF16 led to mitochondrial dysfunction, as evidenced by the increased mtDNA leakage from mitochondria (Figure 6C), the lower ATP level (Figure 6D) and enhanced ROS production (Figure 6B) in Foxo1-KO BMMs. In addition, KLF16-knockdown markedly aggravated the expressions expression of pro-inflammatory *IL-1β* (Figure 6E) and *iNOS* (Figure 6B) in *Foxo1^M-KO^* BMMs.

Next, we explored the effects of KLF16 on mitochondrial biogenesis and inflammatory response in Nrf1-KO macrophages. Nrf1-deficient BMMs were transfected with KLF16-activation plasmids and stimulated by LPS. Indeed, KLF16-activation led to TFAM protein increase, whereas the expression of CytC was suppressed in Nrf1-deficient BMMs (Figure 6F). In particular, the amount of TFAM in mitochondria was enhanced by KLF16-activation plasmids (Figure 6G). Next, we determined the role of KLF16 in mitochondrial function and macrophage damage in Nrf1-deficient BMMs. The elevation of mtDNA leakage (Figure 6H) and ROS production (Figure 6G) was reversed by KLF16-activation plasmids, accompanied by restored ATP production compared to Nrf1-KO cells in the control group (Figure 6I). Moreover, KLF16 activation also abolished Nrf1-KO induced pro-inflammatory IL-1β release (Figure 6J) and macrophages M1 polarization (Figure 6G). These results validate that the mitochondrial functions of KLF16 are crucial to Nrf1-Foxo1 axis regulated M1 polarization and inflammation.

### TFAM is crucial to maintain mitochondrial biogenesis and engage anti-inflammatory innate immunity

**Figure 7:**
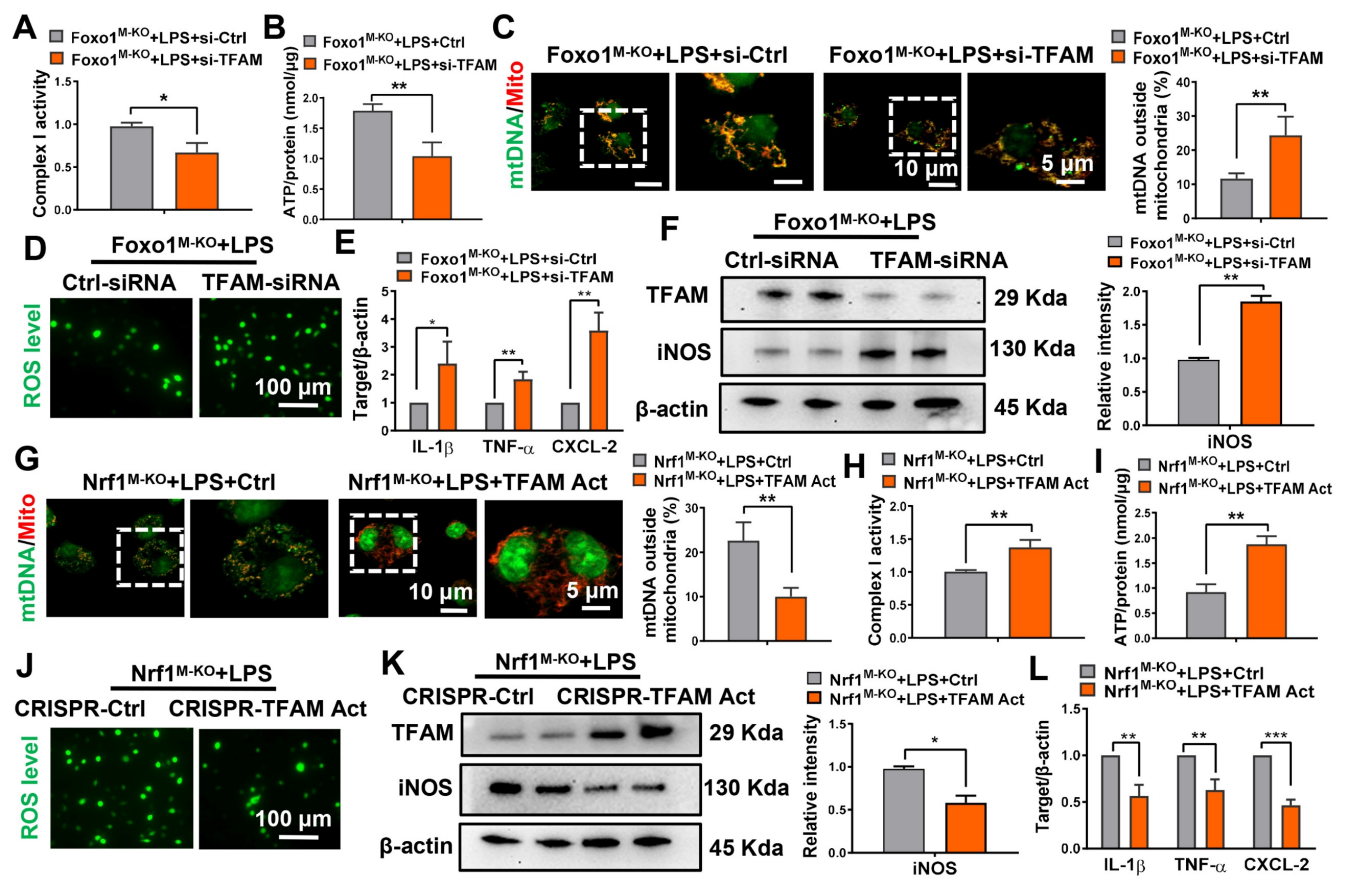
** TFAM is crucial to maintain mitochondrial biogenesis and engage anti-inflammatory innate immunity.** Bone marrow-derived macrophages (BMMs) derived from *Foxo1^M-KO^* or *Nrf1^M-KO^* mice were subjected to transfection with TFAM-siRNA, TFAM-activation plasmids and treated with LPS for 6h. Measurement of mitochondrial respiratory complex I activity (A, H), ATP level (B, I) in BMMs, N = 4/group; The colocalization analysis of cytosol mtDNA (Picogreen) released from mitochondria (MitoTracker Red) in BMMs, Scale bars: 10 μm, 5 μm, N = 4/group (C, G); (D, J) DCFH-DA fluorescence staining of BMMs. Scale bars: 20 μm, N = 4/group; (E, K) qRT-PCR was used to measure the levels of IL-1β, TNF-α, and CXCL-2 in macrophages, N = 6/group; (F, L) Western-assisted analysis of TFAM and iNOS, N = 6/group. Error bars depict mean ± standard error of the mean (SEM), *p < 0.05, **p < 0.01, ***p < 0.001.

To further test the functional role of TFAM in the regulation of mitochondrial biogenesis and innate immunity, BMMs were isolated from the *Foxo1^M-KO^* and *Nrf1^M-KO^* mice. Indeed, siRNA-mediated TFAM knockdown exacerbated mitochondrial damage in *Foxo1^M-KO^* macrophages treated with LPS, as evidenced by the decrease of ATP production (Figure 7A) and mitochondrial respiratory complex I activity (Figure 7B), and the increase of mtDNA leakage from mitochondria (Figure 7C) and ROS release (Figure 7D). In addition, we observed that TFAM-knockdown augmented pro-inflammatory cytokines expression (Figure 7E) and iNOS protein level (Figure 7F) in macrophages. However, TFAM overexpression restored mitochondrial function (Figure 7G-J) and inhibited pro-inflammatory M1 polarization (Figure 7K-L) in *Nrf1^M-KO^* macrophages co-cultured with LPS.

## Discussion

To our knowledge, this is the first study to demonstrate that Nrf1/Foxo1 axis-mediated mitochondrial metabolism regulation is vital for macrophage function modulation in liver fibrosis. The key findings include: (1) Nrf1 expression in macrophages is decreased and negatively correlates with histological damage observed in liver fibrosis; (2) myeloid Nrf1 deficiency aggravates M1 polarization, enhances inflammatory response and facilitates liver fibrosis; (3) Nrf1 directly binds to Foxo1 in the nucleus and inhibits downstream *KLF16* transcription; (4) KLF16 is critical for Nrf1/Foxo1-regulated mitochondrial energy metabolism, macrophage polarization and inflammatory response in fibrotic livers. Our results underscore the critical role of Nrf1 in regulating mitochondrial activity and inflammatory responses in macrophages during the progression of liver fibrosis.

Uncontrolled inflammation has been shown to play a key role in turning self-limiting tissue repair processes into a harmful cycle, which ultimately aggravates liver fibrosis 24.

Macrophages are widely regarded as central contributors to the development of liver inflammation and have a significant impact on both the progression and resolution of liver fibrosis 25. Typically, activated macrophages are categorized into two main subtypes: M1 and M2. M1-polarized macrophages are known to disrupt local homeostasis and contribute to the worsening of fibrosis 26. Nrf1, a nuclear transcription factor, is primarily recognized for its role in stress adaptation during inflammatory responses. Nrf1 is commonly considered a proinflammatory gene, potentially involved in stress adaptation and detoxification 27. Research has demonstrated that somatic inactivation of liver Nrf1 results in nonalcoholic steatohepatitis and hepatic neoplasia 28. However, Nrf1 in macrophages promotes mitochondrial protein turnover via the ubiquitin-proteasome system, which helps alleviate mitochondrial stress and reduces inflammation 29. Our findings suggest that the expression level of Nrf1 is notably reduced within macrophages derived from fibrotic liver tissues in both humans and mice. Additionally, the exacerbation of liver fibrosis, inflammation, and mitochondrial dysfunction occurs specifically upon deletion of Nrf1 in myeloid cells, including Kupffer cells and bone marrow-derived macrophages. Together, these findings strongly support the macrophage-specific nature of Nrf1 in the inflammatory response of macrophages in liver fibrosis progression. Thus, further investigation is necessary to elucidate the mechanisms through which Nrf1 regulates cellular energy metabolism in liver fibrosis progression.

The transcription factor Foxo1 serves various essential functions in regulating innate immune responses under oxidative stress conditions 30. Our earlier research has shown that myeloid Foxo1-β-catenin axis is pivotal in mediating liver inflammation and necroptosis induced by oxidative stress 24. Recent research indicates that Foxo1 signaling in macrophages plays a crucial role in regulating STING-mediated innate immune responses during the progression of MASH 31. Furthermore, hepatocytes Foxo1 is known to mediate fibrosis through upregulating hepatocyte TGF-β1 expression and activating hepatic stellate cells 32. Thus, it is crucial to investigate whether macrophage-specific Foxo1 signaling contributes to liver fibrosis processes potentially through distinct or complementary pathways. Aligning with these findings, we observed that metabolic stress upregulated Foxo1 expression in three different mouse models of liver fibrosis. Additionally, Foxo1 deficiency in macrophages led to reduced ROS production, restored mitochondrial biogenesis, and curtailed inflammation in *Foxo1^M-KO^* macrophages.

Another striking finding was that Nrf1 can translocate into the nucleus and interact with Foxo1 to regulate the transcription of mitochondria biogenesis-related genes. Our in vitro study demonstrated that the nuclear expression of Nrf1 and Foxo1 were increased under LPS stimulation. Importantly, Nrf1 interacted with Foxo1 through direct binding in the nucleus, as confirmed by double immunofluorescence, FRET analyses, GST pull-down and immunoprecipitation assay. Further exploration through ChIP-seq and ChIP-PCR revealed that Foxo1 binds to the *KLF16* promoter to repress its transcription, a role confirmed by the yeast-one-hybrid assay. While Nrf1 does not directly bind to the *KLF16* promoter region. Additionally, functional studies demonstrated that Nrf1 deficiency diminishes KLF16 transcripts, indicating that Nrf1 is required for maintaining Foxo1-mediated regulation of KLF16 expression. Together, these results support a model in which Nrf1 interacts with Foxo1 to facilitate its binding to the *KLF16* promoter and modulate *KLF16* transcription. While our current data underscores this functional partnership, additional studies, such as structural or biophysical analysis of the Nrf1-Foxo1 interaction, would provide deeper mechanistic insights.

KLFs belong to a member of zinc finger-containing transcriptional factors, which are involved in growth, development and metabolism homeostasis 33-34. Numerous studies have reported that KLF9, KLF10 and KLF15 can regulate hepatic glucose homeostasis by increasing the activity of peroxisome proliferator-activated receptor-γ coactivator-1 alpha 35-36. Recently, KLF6 was identified as a novel regulator of autophagy and acted as a new target for graft protection following liver transplantation 37. As one of the most well-known KLFs, KLF16 coordinates multiple liver pathological processes including hepatic lipid metabolism and insulin response 38. Consistent with these results, we offer evidence that Foxo1 suppresses KLF16 expression by directly interacting with the *KLF16* promoter. The upregulated transcription of *KLF16* promotes mitochondrial biogenesis in macrophages.

TFAM is among the most abundant mitochondrial DNA-binding proteins that regulate mtDNA transcription and packaging 39. Tissue-specific absence of TFAM impairs OXPHOS, leads to genetic mitochondrial diseases in both humans and mice 40-41. Previous studies have proved that TFAM serves as a critical regulator in both KLF16 signaling and Nrf1 signaling. A specific and direct binding of KLF16 to the BTE region in *TFAM* promoter has been demonstrated by chromatin immunoprecipitation assay in glioma cells 42. In alcohol-induced hepatic injury model, Nrf1 silencing produced a 1:1 knockdown of TFAM expression and mtDNA depletion 43. Therefore, we hypothesize that KLF16 modulates liver inflammation via the regulation of TFAM-mediated mitochondrial function. As expected, we found that the ablation of KLF16 inhibited TFAM expression accompanied by impaired mitochondrial function but augmented iNOS level in LPS-stimulated *Foxo1^M-KO^* macrophages. However, KLF16 overexpression can positively regulate mitochondrial TFAM level and diminish mtDNA leakage in LPS-stimulated *Nrf1^M-KO^* macrophages. Consistent with this finding, disruption of TFAM led to mtDNA content depletion, complex I activity reduction but promoted macrophage M1 polarization. Finally, TFAM overexpression in macrophages markedly ameliorated mitochondrial dysfunction and inflammatory response. Taken together, these results reveal that KLF16-TFAM axis mechanistically links to mitochondria stress-dependent innate immune regulation. However, how TFAM promotes the mtDNA stability in liver macrophages is still unknown.

## Conclusions

**Figure 8:**
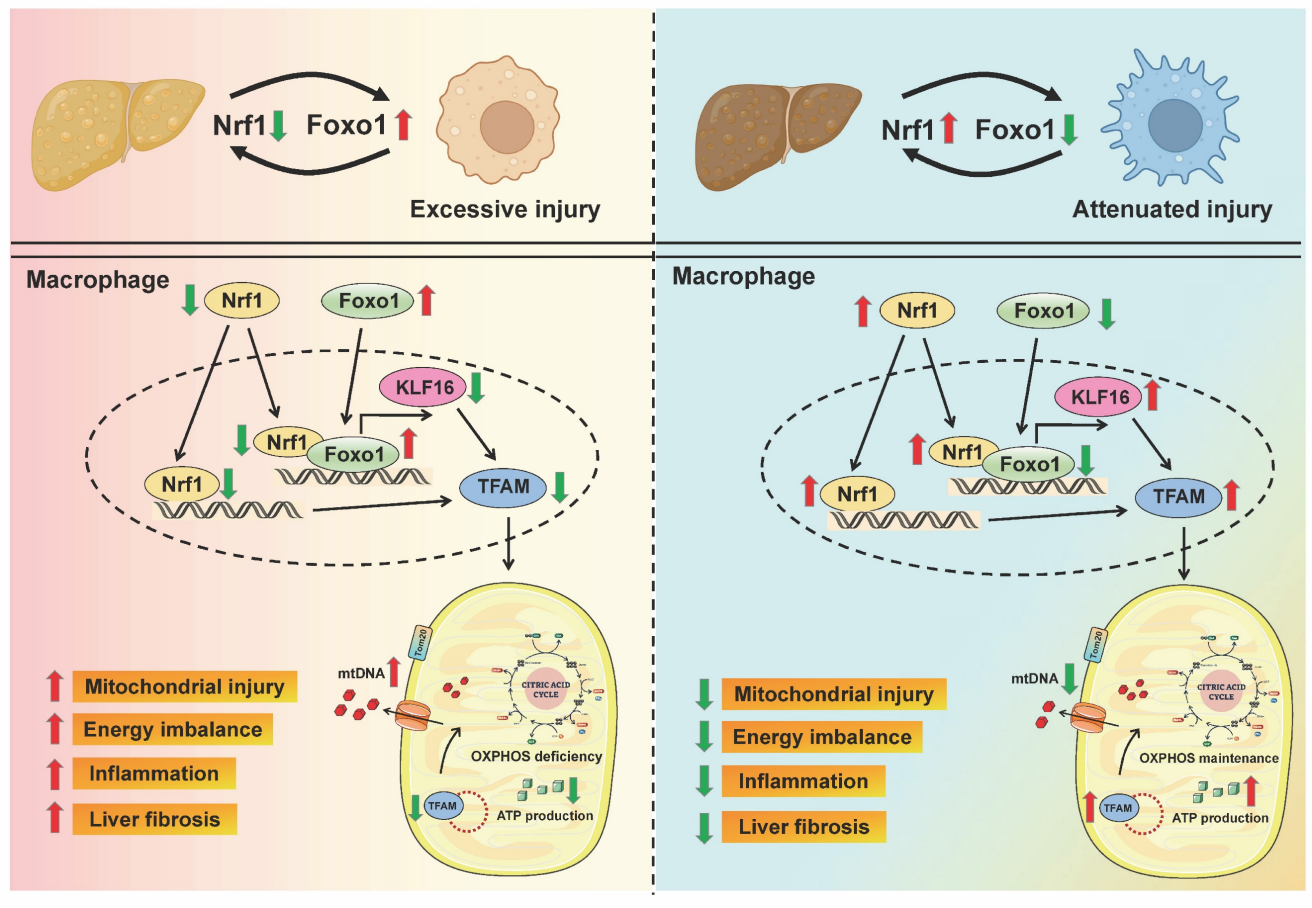
** Schematic summary of this study.** Our findings demonstrate that macrophage Nrf1 deficiency mediates liver fibrosis by impairing mitochondrial biogenesis and respiratory function. Mechanistically, Nrf1 acts as a transcriptional corepressor of Foxo1 through a direct interaction. The target gene KLF16 regulated by the Nrf1-Foxo1 complex is crucial for the modulation of mitochondrial function and immune response.

In summary, our study elucidates the functional roles of macrophage Nrf1-Foxo1 signaling in the pathogenesis of liver fibrosis. Our findings demonstrate that macrophage Nrf1 deficiency mediates liver fibrosis by impairing mitochondrial biogenesis and respiratory function. Mechanistically, Nrf1 acts as a transcriptional corepressor of Foxo1 through a direct interaction. The target gene KLF16 regulated by the Nrf1-Foxo1 complex is crucial for the modulation of mitochondrial function and immune response (Figure 8). By elucidating the molecular pathways through which the Nrf1-Foxo1 axis regulates liver inflammation, our finding provides a basis for developing innovative therapeutic strategies targeting macrophage-driven liver inflammation and fibrosis.

## Supplementary Material

Supplementary methods, figures and tables.

## Abbreviations

DAMPs: danger-associated molecular patterns
HSCs: hepatic stellate cells
ECM: extracellular matrix
OXPHOS: oxidative phosphorylation
Nrf: nuclear factor erythroid 2-like factor
ER: endoplasmic reticulum
DDI2: DNA damage inducible 1 homolog 2
TFAM: mitochondrial transcription factor A
STAT1/3: signal transducers and activators of transcription 1/3
KO: knockout
MASH: metabolic dysfunction-associated steatohepatitis
SPF: specific pathogen-free
HFD: high fat diet
NCD: normal chow diet
CCl_4_: carbon tetrachloride
BDL: bile duct ligation
Foxo1: forkhead box protein o1
M-CSF: macrophage colony-stimulating factor
BMMs: bone marrow-derived macrophages
DMEM: dulbecco's modified eagle's medium
PBS: phosphate -buffered solution
KLF16: kruppel-like factor 16
ANOVA: analysis of variance
SEM: standard error of the mean
qRT-PCR: quantitative real-time reverse transcriptase PCR
ALT: alanine aminotransferase
AST: aspartate aminotransferase
α-SMA: α-smooth muscle actin
TGF-β: transforming growth factor-beta1
Col1a1: collagen type I α1 chain
TIMP1: tissue inhibitor of metalloproteinase 1
Col3a1: collagen type III α1 chain
TNF-α: tumor necrosis factor-α
IL-1β: interleukin-1β
TEM: transmission electron microscopy
iNOS: inducible nitric oxide synthase
Arg1: arginase-1
FRET: fluorescence resonance energy transfer
GST: glutathione-S-transferase
ChIP-Seq: ChIP coupled to massively parallel sequencing
