# Supplementary material for: Macrophage Nrf1/NFE2L1-Foxo1 axis controls liver fibrosis by modulation of mitochondrial reprogramming
Source: Theranostics. 2026 Jan 1;16(5):2269–83. doi: 10.7150/thno.112337 (PMC12712952; doi:10.7150/thno.112337)

Supplementary Materials

Macrophage Nrf1/NFE2L1-Foxo1 axis controls liver fibrosis by modulation of mitochondrial reprogramming

Yuanbang Lin[1#*], Xiyun Bian[3#], Yao Yao[4], Jingmai Xu[5], Yingli Cao[2], Qiong Wu[4], Wen Ning[6], Lian Li[6], Mingwei Sheng[2*], Fengmei Wang[4*].

[1]Department of General Surgery, Tianjin Medical University General Hospital, Tianjin 300052, China.

[2]Department of Anesthesiology, Tianjin First Central Hospital, Tianjin 300192, China.

[3]Central Laboratory, Tianjin Fifth Central Hospital, Tianjin 300450, China.

[4]Department of Hepatology and Gastroenterology, Tianjin First Central Hospital, Tianjin 300192, China.

[5]School of Public Health, North China University of Science and Technology, Tangshan 063000, China.

[6]College of Life Sciences, State Key Laboratory of Medicinal Chemical Biology, Nankai University, Tianjin 300071, China.

[#] These authors contributed equally to this work.

***Corresponding author**: Yuanbang Lin, MD, PhD., Department of General Surgery, Tianjin Medical University General Hospital, Anshan Road NO. 154, Tianjin, PR China, 300052. Email: linyuanbang@tmu.edu.cn. Mingwei Sheng, Department of Anesthesiology, Tianjin First Central Hospital, Fukang Road NO. 24, Tianjin, PR China, 300192. Email: wangfengmei@tmu.edu.cn. Fengmei Wang, MD, PhD., Department of Hepatology and Gastroenterology, Tianjin First Central Hospital, Fukang Road NO. 24, Tianjin, PR China, 300192. Email: wangfengmei@tmu.edu.cn.

Table of Contents

Supplementary Materials and Methods ……………………………………..3

Table S1 ……………………………………………………………………10

Table S2 ……………………………………………………………………11

Table S3 ……………………………………………………………………12

Figure S1 ……………………………………………………………………..15

Figure S2……………………………………………………………...……...16

Figure S3……………………………………………………………………17

Figure S4……………………………………………………………………18

Figure S5……………………………………………………………………20

Figure S6……………………………………………………………………21

Figure S7……………………………………………………………………22

Figure S8……………………………………………………………………23

Figure S9……………………………………………………………………24

Figure S10 …………………………………………………………………25

Figure S11 …………………………………………………………………26

Uncropped WB images……………………………………………………27

Supplementary Materials and Methods

Isolation of Kupffer cells and hepatocytes

Primary liver macrophages (Kupffer cells) and hepatocytes were isolated using a previously described method [1]. Briefly, the liver of the mouse was subjected to in situ digestion at 37 °C using 1 mmol/L EGTA, followed by treatment with a 0.75 g/L solution of type I collagenase. Viable hepatocytes were then collected through centrifugation and plated in 6-well or 12-well plates. To isolate nonparenchymal cells (NPCs) from the hepatocytes, centrifugation was performed at 50 g for 2 min. The NPCs were then suspended in HBSS and separated by a two-step Percoll gradient method (50%/25%) using centrifugation at 1800 g for 15 min at 4 °C. The Kupffer cells, located in the middle layer of the gradient, were collected, resuspended in DMEM culture medium, and purified by removing non-adherent cells through medium exchanges.

Histology, immunohistochemistry and immunofluorescence (IF) staining

Liver tissues were preserved in 4% formalin for 24 h, embedded in paraffin, and sliced into sections with a thickness of 5 μm. For histopathology, the sections underwent staining with hematoxylin and eosin (H&E) or 0.1% Sirius Red following standard procedures. Immunohistochemistry (IHC) staining involved dehydration of the sections, antigen retrieval, and incubation overnight at 4 °Cwith α-SMA (#19245, Cell Signaling Technology, 1:200 dilution) and Ly-6G (ab261916, Abcam, 1:200 dilution) primary antibodies. Similarly, immunofluorescence (IF) staining was conducted on tissue sections or cultured cells that had been fixed in 4% formalin for 30 min, followed by exposure to antibodies at 4 °C overnight. Antibodies used included F4/80 (#30325, Cell Signaling Technology, 1:100 dilution), CD11b (ab184308, Abcam, 1:100 dilution), CD68 (#26042, Cell Signaling Technology, 1:100 dilution), Nrf1 (ab175932, Abcam, 1:100 dilution), Foxo1 (#2880, Cell Signaling Technology, 1:100 dilution), TFAM (#15218, Cell Signaling Technology, 1:100 dilution), iNOS (#13120, Cell Signaling Technology, 1:100 dilution), HNF-4α (ab200142, Abcam, 1:200 dilution), and Tom20 (#42406, Cell Signaling Technology, 1:200 dilution). Alexa Fluor 488- or Alexa Fluor Cy5-conjugated secondary antibodies (Jackson ImmunoResearch) were

subsequently kept in the dark at room temperature for 2 h. To visualize mtDNA, BMMs were incubated with PicoGreen (Invitrogen, P11495) for 1 h and MitoTracker Red (Invitrogen, M7512) for 30 min at 37 ℃. Keyence BZ-X810 fluorescence microscope (Osaka, Japan) was used to capture fluorescence images. ImageJ software was used to analyse the colocalization of green and red fluorescence. Histological fibrosis was detected by Masson's, Sirius red and α-SMA staining. The positive area was quantified using Image J software. Fibrosis was scored , and three randomly selected nonoverlapping fields were used for pathological scoring according to the Ishak scoring system.

Fluorescence resonance energy transfer (FRET) analysis

Fluorescence resonance energy transfer (FRET), a powerful method to study protein interactions in living cells, refers to the transfer of energy from a donor fluorophore to an acceptor, which occurs if the emission spectrum of the donor exhibits overlap with the absorption spectrum of the acceptor. For data acquisition, the donor channel was excited at 458 nm, and the emission was detected at 475-525 nm. The acceptor channel was excited at 514 nm, and its emission was detected at 545-600 nm. The FRET channel was excited at 458 nm, and the emission was detected at 545-600 nm. The FRET signal was corrected with Zen2009 software for substrate donor and acceptor.

Quantitative real-time PCR

RNA extraction was performed on liver tissues or primary cells, followed by reverse transcription utilizing TaqMan Reverse Transcription Reagents (15596026, Invitrogen). Then cDNA was synthesized according to the PrimeScript RT kit (A15300, Invitrogen). SYBR Green PCR Kit (4367659, Applied Biosystems) was employed to perform quantitative PCR (qPCR). Primer sequences were listed in Supplementary Table 3.

Yeast one-hybrid assay

Yeast-one-hybrid assay was used for detection the direct binding of Nrf1 or Foxo1 to KLF16 promoter region. The bait vector pHIS2-KLF16 and prey vector pGADT7-Nrf1 or pGADT7-Foxo1 were co-transformed into Y187 yeast cells. The co-transformants were selected using minimal synthetic defined (SD) medium lacking leucine and tryptophan, while interactions were assessed on SD medium deficient in leucine, tryptophan, and histidine, supplemented with

an appropriate 3-AT concentration. The pHIS2-p53 and pGAD53m constructs were used as positive control.

Protein extraction and western blot

Protein samples were extracted using RIPA lysis buffer (89900, ThermoFisher) and then homogenized on ice for 30 min. Following this, centrifugation at 12,000g for 30 min was performed to collect the supernatant. A total of 40 μg of protein per sample was resolved via SDS-PAGE (sodium dodecyl sulfate-polyacrylamide gel electrophoresis) and subsequently transferred onto a nitrocellulose membrane. Western blot assay was performed with antibodies including Nrf1 (ab175932, Abcam, 1:1000 dilution), Foxo1 (#2880, Cell signaling Technology, 1:1000 dilution), KLF16 (Bioss, bs-16755R, 1:1000 dilution), TFAM (#15218, Cell signaling Technology, 1:1000 dilution), iNOS (#13120, Cell signaling Technology, 1:1000 dilution), SOD2 (#13141, Cell signaling Technology, 1:1000 dilution), Cytochrome C (#11940, Cell signaling Technology, 1:1000 dilution), Lamin B2 (#13823, Cell signaling Technology, 1:1000 dilution), β-actin (#4970, Cell signaling Technology, 1:1000 dilution), anti-rabbit IgG (#7074, Cell signaling Technology, 1:2000 dilution), and anti-mouse IgG (#7076, Cell signaling Technology, 1:2000 dilution). Protein expression was analyzed using the iBright FL1000 imaging system (Invitrogen), with β-actin serving as the normalization control.

CRISPR activation plasmids

CRISPR activation plasmids facilitate targeted gene identification and upregulation by leveraging a deactivated Cas9 (dCas9) nuclease, featuring D10A and N863A mutations, fused to the VP64 activation domain. This system operates in tandem with a target-specific single-guide RNA (sgRNA) engineered to interact with the MS2-P65-HSF1 fusion protein. The synergy of this synergistic activation mediator (SAM) transcription activation platform ensures efficient enhancement of endogenous gene expression. The KLF16 (TFAM) CRISPR activation plasmids (m) include three components in a 1:1:1 mass ratio: (1) the CRISPR/dCas9-VP64-Blast plasmid, which encodes the dCas9-VP64 fusion protein along with a blasticidin resistance gene; (2) the MS2-P65-HSF1-Hygro plasmid, which encodes the MS2-P65-HSF1 fusion protein and a hygromycin resistance gene; and (3) the sgRNA (MS2)-Puro

plasmid, which provides a unique, target-specific 20-nucleotide guide RNA and a puromycin resistance gene. Together, these plasmids form the SAM complex, a highly effective transcriptional activation system designed to upregulate KLF16 (TFAM).

Co-immunoprecipitation

Cells were disrupted using NP-40 lysis buffer, and the resulting total extracts were incubated overnight at 4°C with antibodies against either Foxo1 (#2880, Cell Signaling Technology) or Nrf1 (ab175932, Abcam). Protein G/A beads were then added, and the mixture was incubated for 4 h at 4°C. After centrifugation, The pellet was resuspended and heated at 95°C for 5 min to facilitate elution. The collected supernatant was subsequently analyzed using conventional immunoblotting methods.

Reactive oxygen species (ROS) assay

The generation of ROS in macrophages was assessed utilizing H2DFFDA (D399, Invitrogen) following the guidelines provided by the manufacturer. Green fluorescent-positive labeled cells were counted blindly across 10 high-power fields (HPF) for each section.

Mitochondrial membrane potential analysis

The mitochondrial membrane potential ($\Delta\Psi m$) was assessed using JC-1 (Beyotime, China). BMMs were treated with JC-1 (500 nM) in standard DMEM without FBS for 30 min. Fluorescence variations were observed using a laser scanning confocal microscope (Olympus, FV1200). JC-1 monomer green fluorescence was excited at 488 nm with a helium-neon laser and captured through a 525 nm long-pass filter, while JC-1 aggregate red fluorescence was excited at 543 nm and detected via a 590 nm long-pass filter.

Transmission electron microscopy (TEM)

Transmission electron microscopy (TEM) of liver tissue was performed according to the manufacturer's instructions. The sections were stained with 0.3% lead citrate and subsequently imaged using a HITACHI electron microscope (Tokyo, Japan).

RNA-sequencing assay

RNA sequencing was conducted on liver tissues obtained from mouse liver fibrosis models (n = 3/group). Total RNA was isolated using the TRIzol

reagent (15596018, Invitrogen). cDNA libraries were prepared according to the instructions provided by the manufacturer with the UltraTM RNA Library Prep Kit for Illumina (New England Biolabs). All bioinformatics analyses were carried out within the R environment (version 4.0.2). To identify key pathways associated with differentially expressed genes, pathway enrichment analysis was performed using the Kyoto Encyclopedia of Genes and Genomes (KEGG) database. The raw set of RNA-seq data is available in BIG Submission datasets (Accession number: subSAM146519).

Chromatin immunoprecipitation (ChIP)

ChIP analysis was carried out with a ChIP Assay Kit (Abcam) [2]. To briefly summarize, BMMs were fixed with 1% formaldehyde for 10 min to cross-link chromatin and associated proteins, and the reaction was quenched by adding 0.125M glycine for 5 min. The cells were rinsed with ice-cold PBS and lysed in ChIP lysis buffer for 10 min. The nuclei were separated by centrifugation, resuspended in nuclear lysis buffer, and subjected to sonication for 15 min. The size of DNA fragments in purified chromatin was assessed on a 1.5% agarose gel. The sheared chromatin was immunoprecipitated overnight using Foxo1 (#2880, Cell Signaling Technology) antibodies. Normal IgG served as a control in place of Foxo1 antibody. Antibody-bound chromatin was incubated with protein A sepharose beads. Protein-DNA complexes were washed, eluted, and subjected to cross-link reversal. Then the DNA was purified afterward and analyzed by PCR. The primer for the Foxo1-responsive region of *KLF16* promoter: forward: 5'- CCCTTGATCGAGTTGCAGGT -3', reverse: 5'- GAACCCCTAGCCTTTGTGCT -3'.

ChIP-sequencing (ChIP-seq)

The ChIP-DNA was processed to create a sequencing library through a series of steps, including genome-wide DNA fragmentation, blunt-end repair, A-tailing, adaptor attachment, and PCR amplification. Unique adaptors were applied to enable multiplexing of multiple samples in a single sequencing lane. Sequencing was performed on an Illumina HiSeq 3000 platform (Illumina, San Diego, CA) with a 50-cycle single-read strategy at the Technology Center for Genomics & Bioinformatics (TCGB). Data quality evaluation was conducted using Illumina SAV, and sample demultiplexing was executed with the Bcl2fastq2 v2.17 software. Reads were aligned to the mouse mm10 genome

via Bowtie1, and peaks were called using MACS2. Peak annotation was conducted using ChIPseeker. Genome browser representation files were created by converting ChIP-seq data into the bigWig format. This conversion involved using genomeCoverageBed from bedtools v2.17.0 to generate a bed file, followed by the UCSC bedGraphToBigWig tool to convert the bed file to bigWig format [3]. The ChIP-seq data has been deposited in BIG Sub with the accession number subPRO06493.

RNA *in situ* hybridization.

RNA in situ hybridization (ISH) was conducted using the RNAscope 2.5 HD Assay-RED KIT (324510, Advanced Cell Diagnostics, CA) as per the manufacturer's instructions [3]. Probes targeting mouse *KLF16*, as well as the corresponding positive and negative controls, were purchased from Advanced Cell Diagnostics. Briefly, BMMs were placed on slides and fixed in 10% neutral buffered formalin, and dehydrated stepwise with ethanol at concentrations of 50%, 70%, and 100% for 5 min each. Following a 5-min period of air-drying, the slides were sequentially incubated with hydrogen peroxide for 10 min and protease IV for 30 min. After washing, the slides were incubated at 40°C with *KLF16*, negative, and positive control probes for 2 h in a HybEZ oven (321710, Advanced Cell Diagnostics, CA, USA). Amplification reagents AMP1-6 were applied in sequence at 40°C with durations of 30, 15, 30, 15, 30, and 15 min, respectively. Slides were rinsed twice with wash buffer between AMP incubations. Subsequently, Fast Red was applied for 10 min to visualize RNA signals, followed by counterstaining with hematoxylin. Slides were dried for 15 min at 60°C before mounting. Light microscopy images of RNA-ISH staining were captured with a Keyence BZ-X810 microscope.

Supplementary Table 1. Baseline characteristics of included patients.

Fibrosis stage	Normal histology	Mild fibrosis (12)			Advanced fibrosis (8)		
		HBV (1~4)	HCV (1~2)	MASH (1~2)	HBV (5~6)	HCV (5~6)	MASH (3~4)
Patients number	10	HBV (3)	HCV (5)	MASH (4)	HBV (1)	HCV (1)	MASH (6)
Age (age)	50.7±9.0	56.7±2.5	52.6±9.6	53±4.5	57	52	55.5±3.7
Serum TB (umol/L)	10.4±2.3	16±2.6	15.4±1.5	13.75±1.3	22.4	20.1	19.4±2.7
Serum ALT (U/L)	21.7±4.8	55.3±5.0	55±5.2	61±7.9	100.2	92.3	103.2±12.5
Serum AST (U/L)	26.5±5.5	62±2.6	59.6±7.1	52±6.7	80.2	78.5	87±10.3
Serum ALB (g/L)	38.3±1.4	38.7±1.5	37.2±1.6	38.3±1.7	36.7	38.2	37.2±1.3

For HBV infection, the Ishak fibrosis score was used, categorizing 1-4 as Mild fibrosis and 5-6 as Advanced fibrosis [4-5]. In HCV infection, the Metavir score was applied, with stages 1-2 classified as Mild fibrosis and 3-4 as Advanced fibrosis [6-7]. For patients with MASH, the NAFLD activity score identified 1-2 as Mild fibrosis and 3-4 as Advanced fibrosis [8-9]. Quantitative variables are presented as mean ± standard deviation (SD). HBV, Hepatitis B virus; HCV, Hepatitis C virus; MASH, metabolic dysfunction-associated steatohepatitis; NAFLD, non-alcoholic fatty liver disease; TB, total bilirubin; ALT, alanine aminotransferase; AST, aspartate aminotransferase; ALB, albumin.

Supplementary Table 2. Formulation of high-fat diet fed to male C57 mice

Ingredient	Grams	Ingredient	Grams
Gluta	38.2	Histidine, L, HCl,	4.60 g
Prolin	17.8	Cystine, L	4.20 g
Leucine, L	15.80 g	Glycine	3.00 g
Lysine, L, HCl	13.20 g	Tryptophan, L	2.10 g
Aspartic Acid, L	12.10 g	Methionine, L	0.80 g
Serine, L	10.00 g	Lodex 10	130.10 g
Vali	9.3	Sucrose, Fine	72.80 g
Tyrosine, L	9.20 g	Solka Floc,	50.00 g
Phen	8.40	Lard	245.00 g
Isole	7.60	Soybean Oil,	25.00 g
Threo	7.20	S10026B	50.00 g
Arginine, L	6.00 g	Sodium	7.50 g
Alanine, L	5.10 g	V10001C	1.00 g

	kcal%
Protein	18.1
Fat	61.6
Carbohydrate	20.3

Supplemental Table 3: Primer sequences for the amplification (H, denotes human and M, denotes mice)

Target genes	Forward primers	Reverse primers
H-Nrf1	5’-GGGCGGGAAGACCTTTTGTA-3’	5’-TCAGTCAGGATCCACTTGCG-3’
H-β-actin	5’-TAAGGAGAAGCTGTGCTACGTC-3’	5’-AGTTTCGTGGATGCCACAGG-3’
M-Nrf1	5’-GGTGGGGGACAGATAGTCCT-3’	5’- GCTGTCCGATATCCTGGTGG-3’
M-IL-1β	5’- TGTAATGAAAGACGGCACACC-3’	5’-TCTTCTTTGGGTATTGCTTGG-3’
M-TNF-α	5’-GCTACCAAACTGGATATAATCAGGA-3’	5’-CCAGGTAGCTATGGTACTCCAGAA-3’
M-CXCL-2	5’-AGGTCCCTGTCATGCTTCTG-3’	5’-TCTGGACCCATTCCTTCTTG-3’
M-TGF-β	5’-TGCGCTTGCAGAGATTAAAA-3’	5’-CTGCCGTACAACTCCAGTGA-3’
M-Col1a1	5’-GCTCCTCTTAGGGGCCACT-3’	5’-CCACGTCTCACCATTGGGG-3’
M-TIMP1	5’-GCAACTCGGACCTGGTCATAA-3´	5’-CGGCCCGTGATGAGAAACT-3´
M-Col3a1	5’-CTGTAACATGGAAACTGGGGAAA-3´	5’-CCATAGCTGAACTGAAAACCACC-3´
M-mtCo1	5’-GACTTGCAACCCTACACGGA -3´	5’-GATGGCGAAGTGGGCTTTTG-3´
M-SDH	5’-TCAGTTCCACCCCACAGGTA-3´	5’-GACATCCACACCAGCGAAGA-3´
M-iNOS	5’-TCACCTGAGCTTTGATGTCG-3´	5’-CTGAAAGGAGCCCTGTCTTG-3´
M-Arg1	5’-TCACCTGAGCTTTGATGTCG-3´	5’-CTGAAAGGAGCCCTGTCTTG-3´
M-TFAM	5’-AACACCCAGATGCAAAACTTTCA-3´	5’-GACTTGGAGTTAGCTGCTCTTT-3´

M-KLF16	5’-GTGTACCAAGCGGTTCACC-3´	5’-CAGGTCGTCGCAGGAGTTC-3´
M-β-actin	5’-GTGACGTTGACATCCGTAAAGA-3’	5’-GCCGGACTCATCGTACTCC-3’

References:

[1] Lin, Y, Sheng, M, Qin, H, et al. Caspase 6 promotes innate immune activation by functional crosstalk between RIPK1-IκBα axis in liver inflammation. Cell Commun Signal. 2023; 21 (1): 282.

[2] Sheng, M, Weng, Y, Cao, Y, et al. Caspase 6/NR4A1/SOX9 signaling axis regulates hepatic inflammation and pyroptosis in ischemia-stressed fatty liver. Cell Death Discov. 2023; 9 (1): 106.

[3] Sheng, M, Lin, Y, Xu, D, et al. CD47-Mediated Hedgehog/SMO/GLI1 signaling promotes mesenchymal stem cell immunomodulation in mouse liver inflammation. Hepatology. 2021; 74 (3): 1560-77.

[4] Sun, Y, Wu, X, Zhou, J, et al. Persistent low level of hepatitis B virus promotes fibrosis progression during therapy. Clin Gastroenterol H. 2020; 18 (11): 2582-91.

[5] Sterling, RK, King, WC, Wahed, AS, et al. Evaluating noninvasive markers to identify advanced fibrosis by liver biopsy in HBV/HIV co-infected adults. Hepatology. 2019; 71 (2): 411-21.

[6] Konerman, MA, Mehta, SH, Sutcliffe, CG, et al. Fibrosis progression in human immunodeficiency virus/hepatitis C virus coinfected adults: prospective analysis of 435 liver biopsy pairs. Hepatology. 2014; 59 (3): 767-75.

[7] Patin, E, Kutalik, Z, Guergnon, J, et al. Genome-wide association study identifies variants associated with progression of liver fibrosis from HCV infection. Gastroenterology. 2012; 143 (5): 1244-52.e12.

[8] Newsome, PN, Sasso, M, Deeks, JJ, et al. FibroScan-AST (FAST) score for the non-invasive identification of patients with non-alcoholic steatohepatitis with significant activity and fibrosis: a prospective derivation and global validation study. Lancet Gastroenterol Hepatol. 2020; 5 (4): 362-73.

[9] Angulo P, Kleiner DE, Dam-Larsen S, et al. Liver fibrosis, but no other histologic features, is associated with long-term outcomes of patients with nonalcoholic fatty liver disease. Gastroenterology. 2015 Aug; 149(2): 389-97.

Figure S1

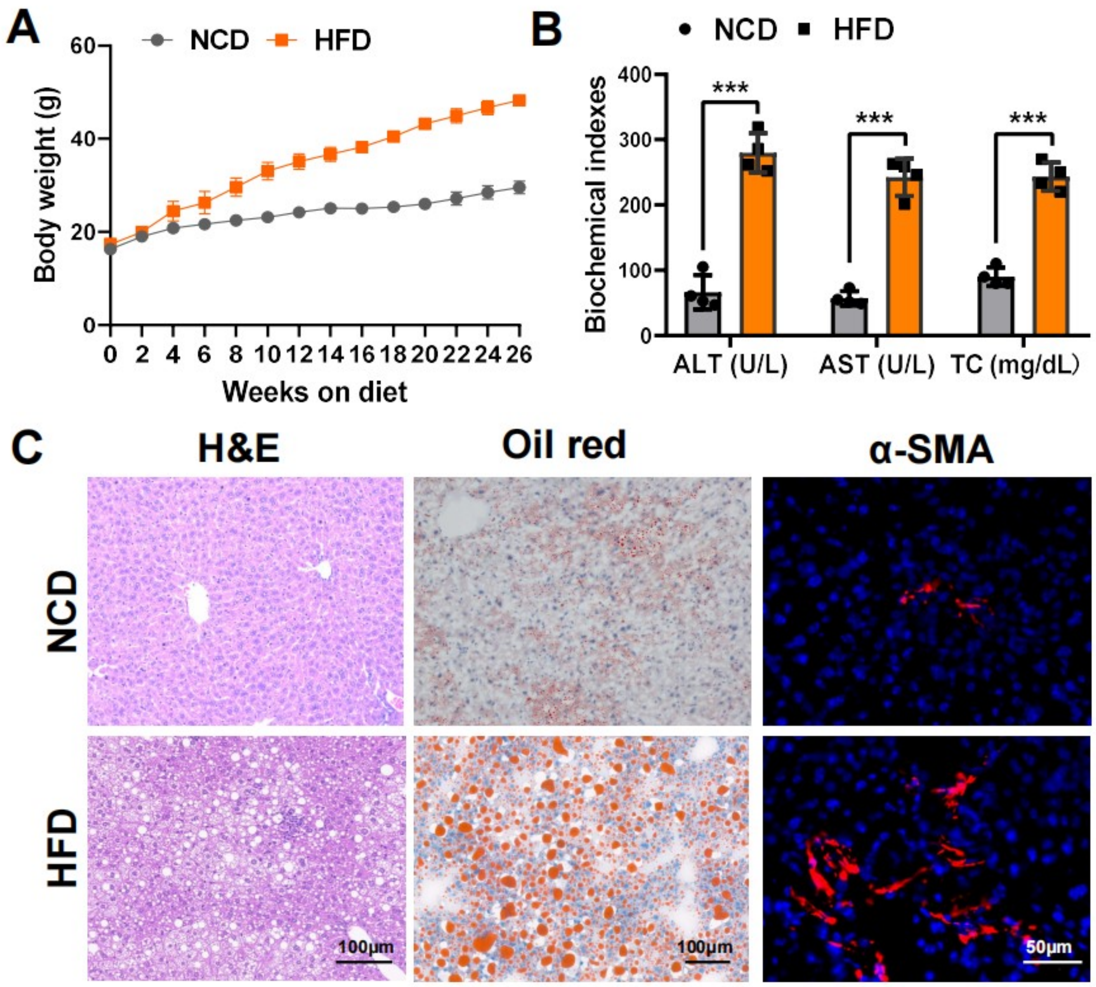

Figure S1. High fat diet feeding for 26 weeks to induce MASH in mice. 4-week-old mice were fed with HFD or NCD for 26 weeks. A, body weight was recorded once every two weeks. B, serum ALT and AST levels (U/L) and TC level (mg/dL) were evaluated. C, Representative histological staining (H&E), Oil Red O staining and Immunofluorescence staining of α-SMA of liver tissues, Scale bars, 100 μm, 50 μm. Analysis of variance (ANOVA), Bonferroni multiple comparison test, N = 4-6 per group. Error bars depict mean ± standard error of the mean (SEM). **p<0.01, ***p<0.001.

Figure S2

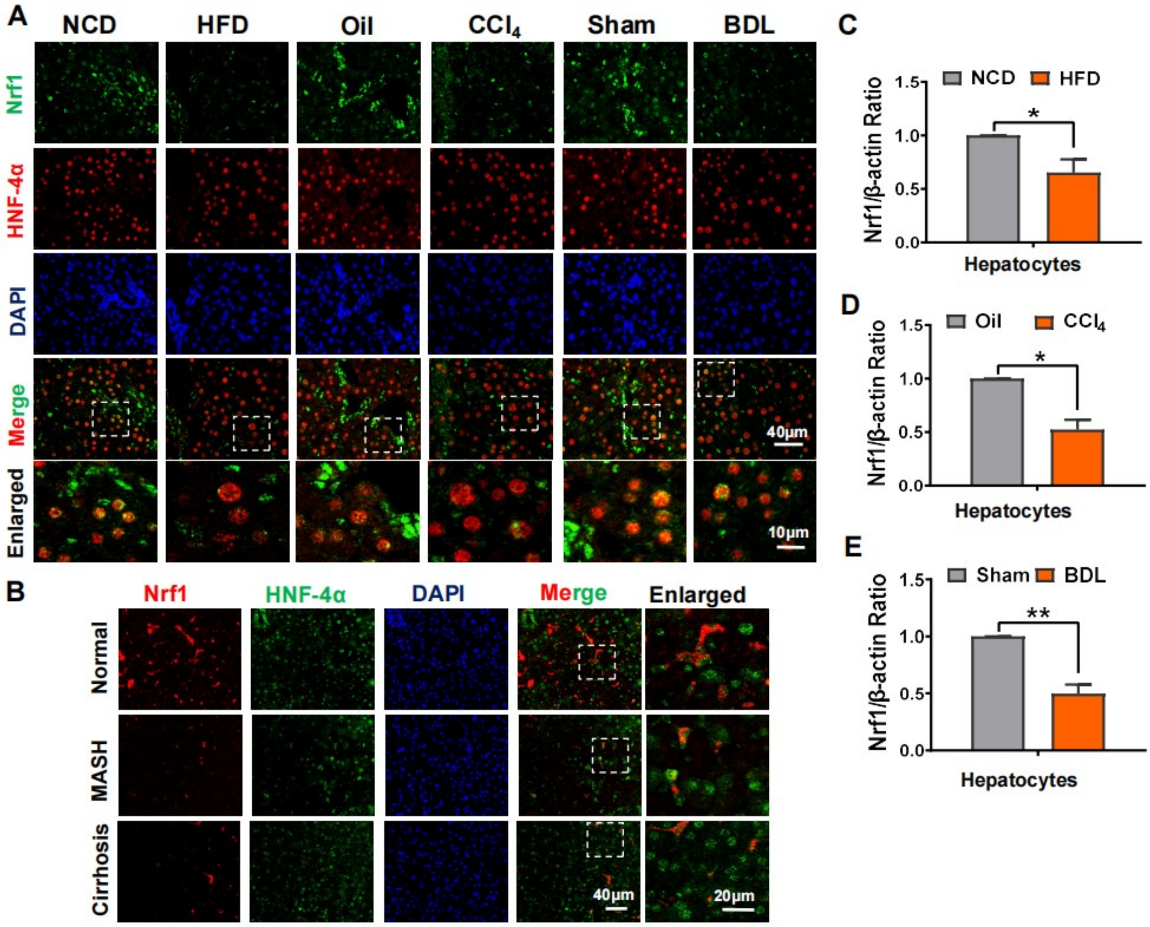

Figure S2. Nrf1 expression is decreased in hepatocytes of liver fibrosis tissues. (A) Dual-immunofluorescence analysis of Nrf1 and HNF-4α in murine liver tissues, scale bars: 40 μm, 10 μm, N = 4/group; (B) Dual-immunofluorescence staining of Nrf1 and HNF-4α in human livers, scale bars: 40 μm, 20 μm, N = 4/group; qRT-PCR analysis of Nrf1 expression in hepatocytes isolated from three mice livers induced by HFD (C), CCl_4 injection (D) and BDL (E), N = 6/group. Error bars depict mean ± standard error of the mean (SEM); *$p < 0.05$, **$p < 0.01$, ***$p < 0.001$.

Figure S3

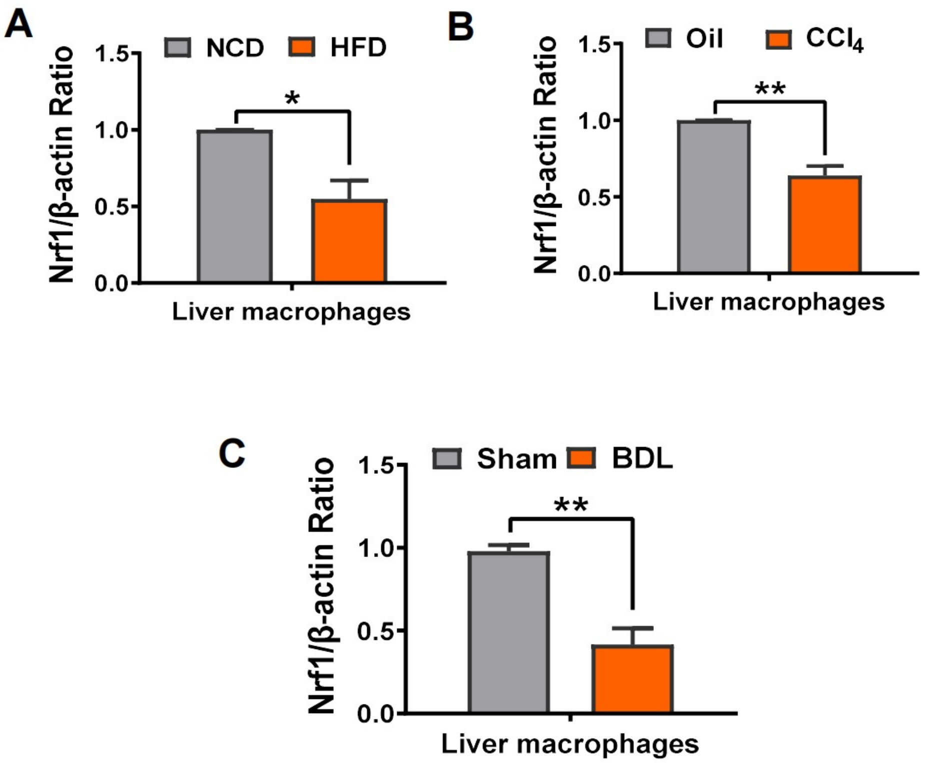

Figure S3. Nrf1 expression is decreased in macrophages of liver fibrosis tissues. qRT-PCR analysis of Nrf1 expression in macrophages isolated from three mice livers induced by HFD (A), CCl_4 injection (B) and BDL (C), N = 6/group. Error bars depict mean ± standard error of the mean (SEM); $^*p < 0.05$, $^{**}p < 0.01$.

Figure S4

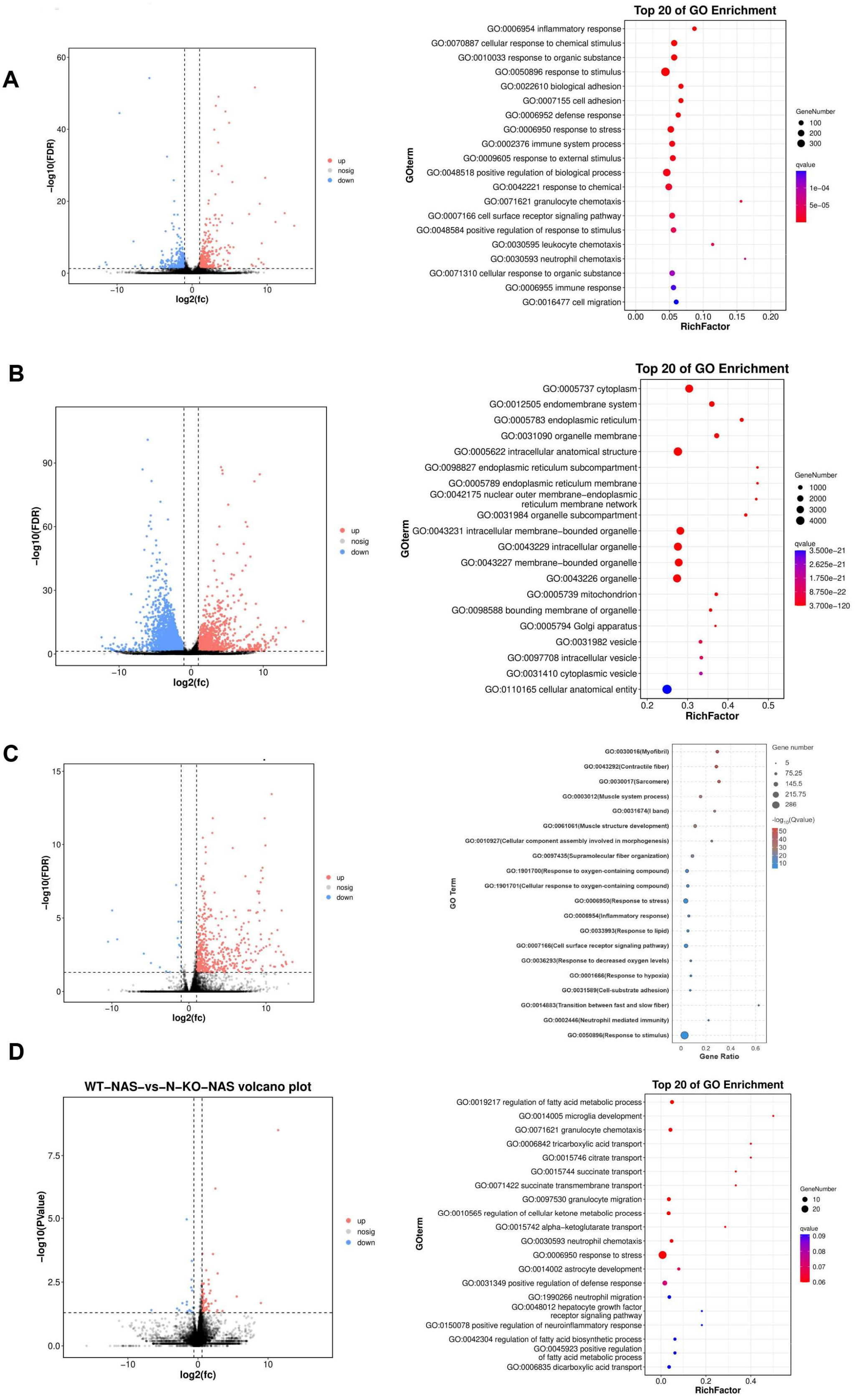
A
-log10(FDR)
log2(fc)
up
nosig
down
Top 20 of GO Enrichment
GO:0006954 inflammatory response
GO:0070887 cellular response to chemical stimulus
GO:0010033 response to organic substance
GO:0050896 response to stimulus
GO:0022610 biological adhesion
GO:0007155 cell adhesion
GO:0006952 defense response
GO:0006950 response to stress
GO:0002376 immune system process
GO:0009605 response to external stimulus
GO:0048518 positive regulation of biological process
GO:0042221 response to chemical
GO:0071621 granulocyte chemotaxis
GO:0007166 cell surface receptor signaling pathway
GO:0048584 positive regulation of response to stimulus
GO:0030595 leukocyte chemotaxis
GO:0030593 neutrophil chemotaxis
GO:0071310 cellular response to organic substance
GO:0006955 immune response
GO:0016477 cell migration
GOterm
RichFactor
GeneNumber
qvalue
B
-log10(FDR)
log2(fc)
up
nosig
down
Top 20 of GO Enrichment
GO:0005737 cytoplasm
GO:0012505 endomembrane system
GO:0005783 endoplasmic reticulum
GO:0031090 organelle membrane
GO:0005622 intracellular anatomical structure
GO:0098827 endoplasmic reticulum subcompartment
GO:0005789 endoplasmic reticulum membrane
GO:0042175 nuclear outer membrane−endoplasmic reticulum membrane network
GO:0031984 organelle subcompartment
GO:0043231 intracellular membrane−bounded organelle
GO:0043229 intracellular organelle
GO:0043227 membrane−bounded organelle
GO:0043226 organelle
GO:0005739 mitochondrion
GO:0098588 bounding membrane of organelle
GO:0005794 Golgi apparatus
GO:0031982 vesicle
GO:0097708 intracellular vesicle
GO:0031410 cytoplasmic vesicle
GO:0110165 cellular anatomical entity
GOterm
RichFactor
GeneNumber
qvalue
C
-log10(FDR)
log2(fc)
up
nosig
down
GO:0030016(Myofibril)
GO:0043292(Contractile fiber)
GO:0030017(Sarcomere)
GO:0003012(Muscle system process)
GO:0031674(I band)
GO:0061061(Muscle structure development)
GO:0010927(Cellular component assembly involved in morphogenesis)
GO:0097435(Supramolecular fiber organization)
GO:1901700(Response to oxygen-containing compound)
GO:1901701(Cellular response to oxygen-containing compound)
GO:0006950(Response to stress)
GO:0006954(Inflammatory response)
GO:0033993(Response to lipid)
GO:0007166(Cell surface receptor signaling pathway)
GO:0036293(Response to decreased oxygen levels)
GO:0001666(Response to hypoxia)
GO:0031589(Cell-substrate adhesion)
GO:0014883(Transition between fast and slow fiber)
GO:0002446(Neutrophil mediated immunity)
GO:0050896(Response to stimulus)
GO Term
Gene Ratio
Gene number
-log10(Qvalue)
D
WT−NAS−vs−N−KO−NAS volcano plot
−log10(PValue)
log2(fc)
up
nosig
down
Top 20 of GO Enrichment
GO:0019217 regulation of fatty acid metabolic process
GO:0014005 microglia development
GO:0071621 granulocyte chemotaxis
GO:0006842 tricarboxylic acid transport
GO:0015746 citrate transport
GO:0015744 succinate transport
GO:0071422 succinate transmembrane transport
GO:0097530 granulocyte migration
GO:0010565 regulation of cellular ketone metabolic process
GO:0015742 alpha−ketoglutarate transport
GO:0030593 neutrophil chemotaxis
GO:0006950 response to stress
GO:0014002 astrocyte development
GO:0031349 positive regulation of defense response
GO:1990266 neutrophil migration
GO:0048012 hepatocyte growth factor receptor signaling pathway
GO:0150078 positive regulation of neuroinflammatory response
GO:0042304 regulation of fatty acid biosynthetic process
GO:0045923 positive regulation of fatty acid metabolic process
GO:0006835 dicarboxylic acid transport
GOterm
RichFactor
GeneNumber
qvalue

Figure S4. Global pathway changes in fibrotic livers. Total RNA from human MASH samples and normal liver samples, three types of fibrotic liver models (MASH, CCl_4 treatment, BDL treatment) from *Nrf1*$^{FL/FL}$ and *Nrf1*$^{M\text{-}KO}$ mice was extracted and subjected to a deep RNA-sequencing (RNA-seq) analysis. The log2 fold changes of gene expression and GO enrichment analysis of transcripts differentially expressed in human MASH samples (A, B); fibrotic livers by BDL treatment (C, D), CCl_4 treatment and HFD feeding (E, F).

Figure S5

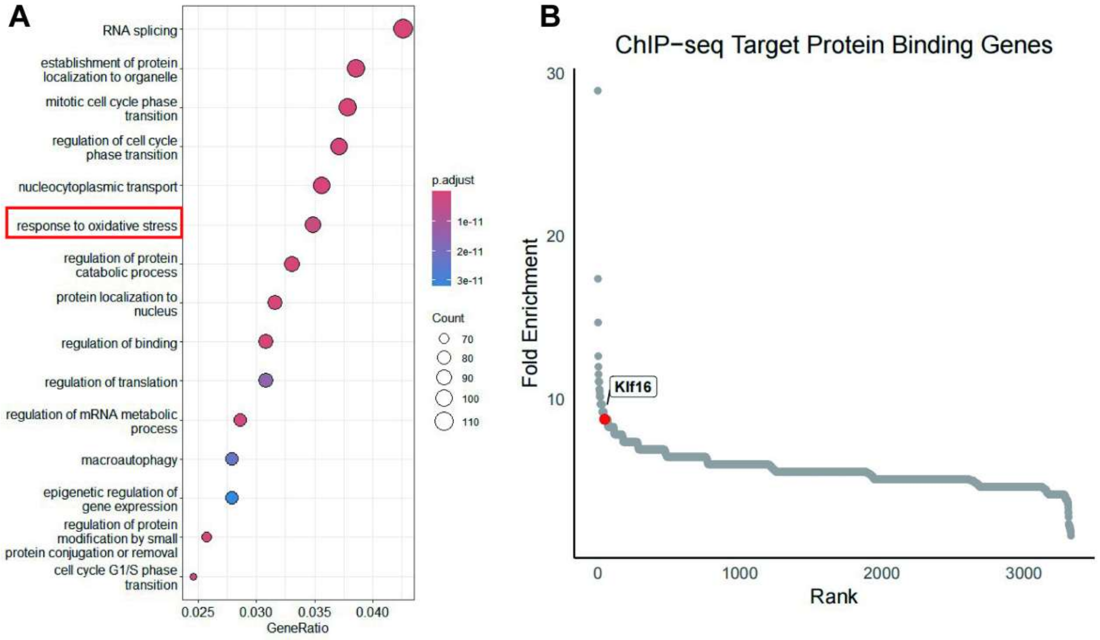

Figure S5. Analysis of all genomic loci that Foxo1 binds to and related pathways. (A) Gene Ontology (GO) analysis of ChIP-seq data for *Foxo1*; (B) *KLF16* was ranked in the Top 50 target genes of Foxo1 in fold enrichment map.

Figure S6

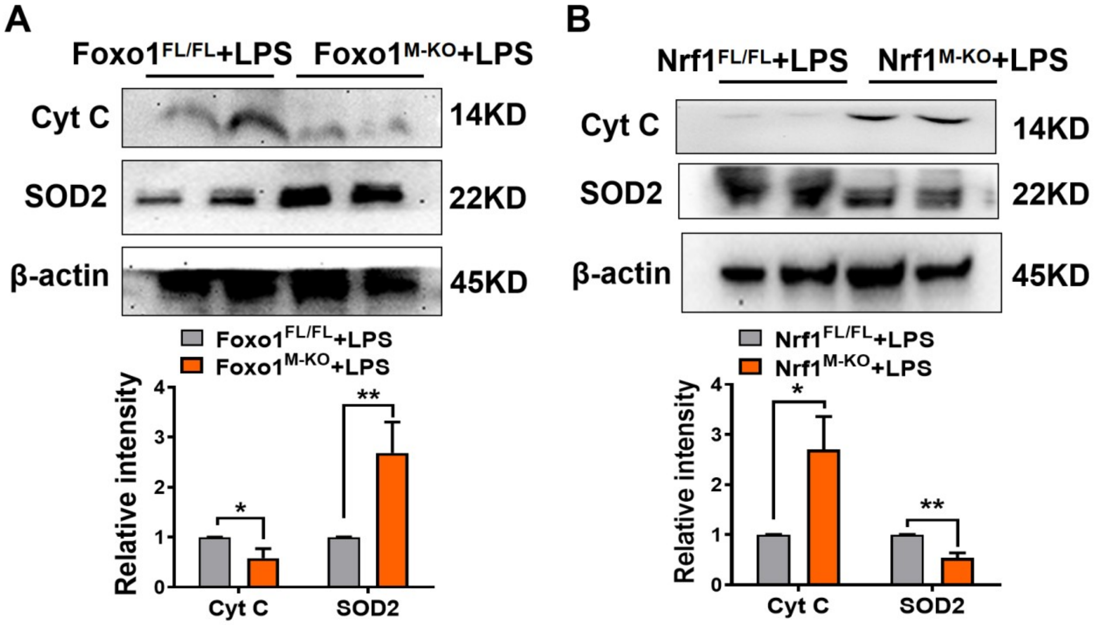

Figure S6 The expression of Cytochrome C (Cyt C) and SOD2 in Bone marrow-derived macrophages (BMMs). BMMs obtained from *Foxo1*$^{M\text{-}KO}$ or *Nrf1*$^{M\text{-}KO}$ mice were treated with LPS for 6 h. (A) Western blot analysis was performed to assess CytC and SOD2 expression in *Foxo1*$^{M\text{-}KO}$ BMMs; (B) Western blot analysis was performed to assess CytC and SOD2 expression in *Nrf1*$^{M\text{-}KO}$ BMMs, N = 6/group. Error bars depict mean ± standard error of the mean (SEM), *p < 0.05, **p < 0.01.

Figure S7

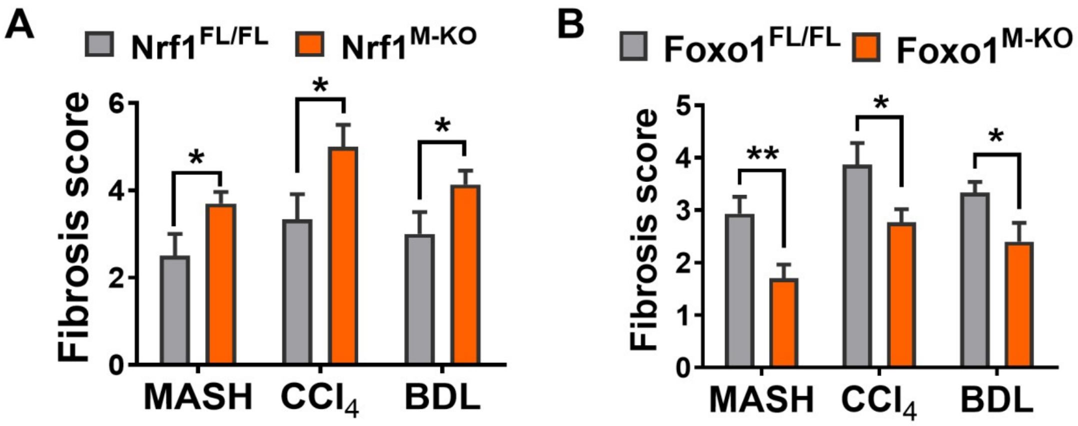

Figure S7. Quantitative analysis of fibrosis score using Ishak scoring system in the *Nrf1$^{M\text{-}KO}$* fibrotic liver samples (A) and the *Foxo1$^{M\text{-}KO}$* fibrotic liver samples, N = 6/group. Error bars represent the mean ± standard error of the mean (SEM); $^{*}p < 0.05$, $^{**}p < 0.01$.

Figure S8

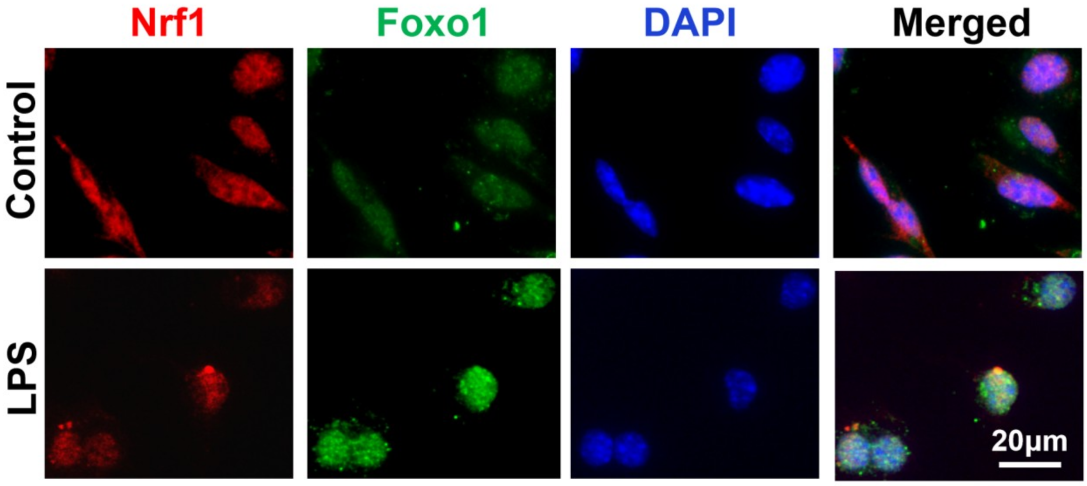

Figure S8. Dual-immunofluorescence staining of Nrf1 and Foxo1 in mice liver samples, scale bar: 20 μm, N = 4/group

Figure S9

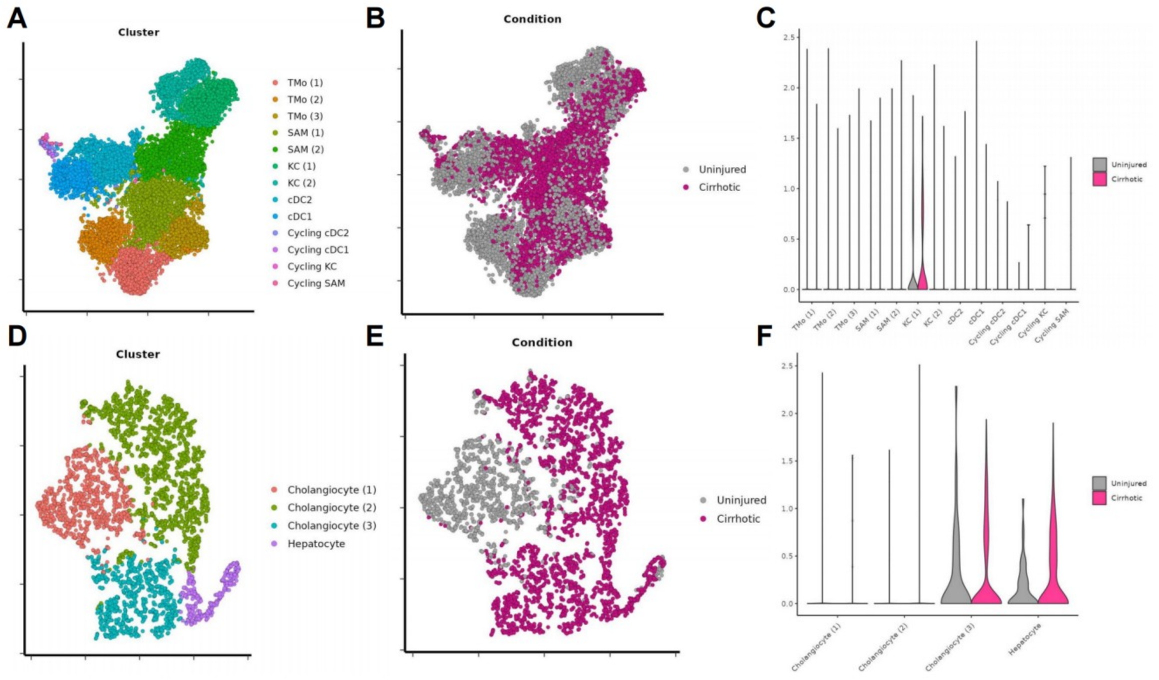

Figure S9. Distribution of Nrf1 in human healthy and fibrotic livers. (A) Clustering and annotating mononuclear phagocytes; (B) Nrf1 gene expression in mononuclear phagocytes; (C) Gene Violin of Nrf1 in mononuclear phagocytes; (D) Clustering and annotating epithelial cells including hepatocytes; (E) Nrf1 gene expression in epithelial cells including hepatocytes; (F) Gene Violin of Nrf1 in epithelial cells including hepatocytes.

Figure S10

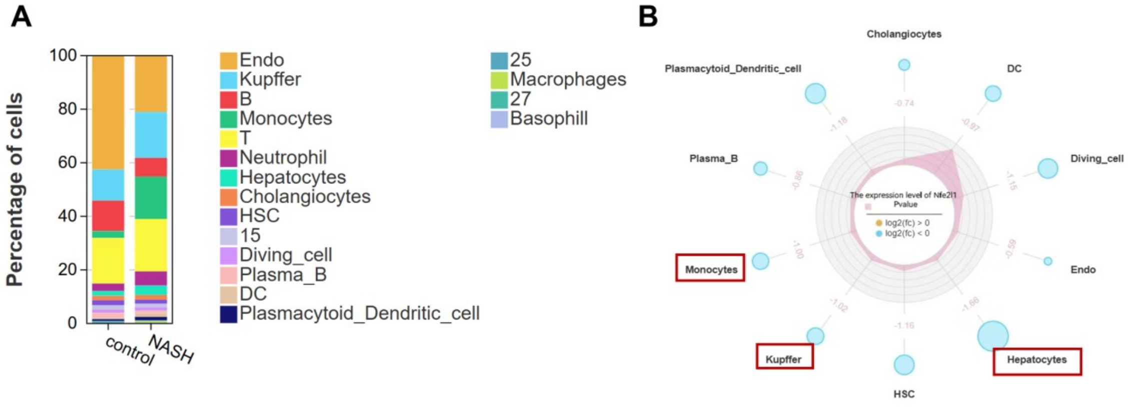

Figure S10. the expression level of Nrf1 in various cell types from mice MASH samples. A. Stacked bar plots illustrating the proportion of distinct cell types in the control and MASH groups; B. Radar plot showing the expression level of Nrf1 in various cell types. The log2 (fold change) values are used to represent the relative expression levels, with log2(fc) > 0 indicating upregulation and log2(fc) < 0 indicating downregulation.

Figure S11

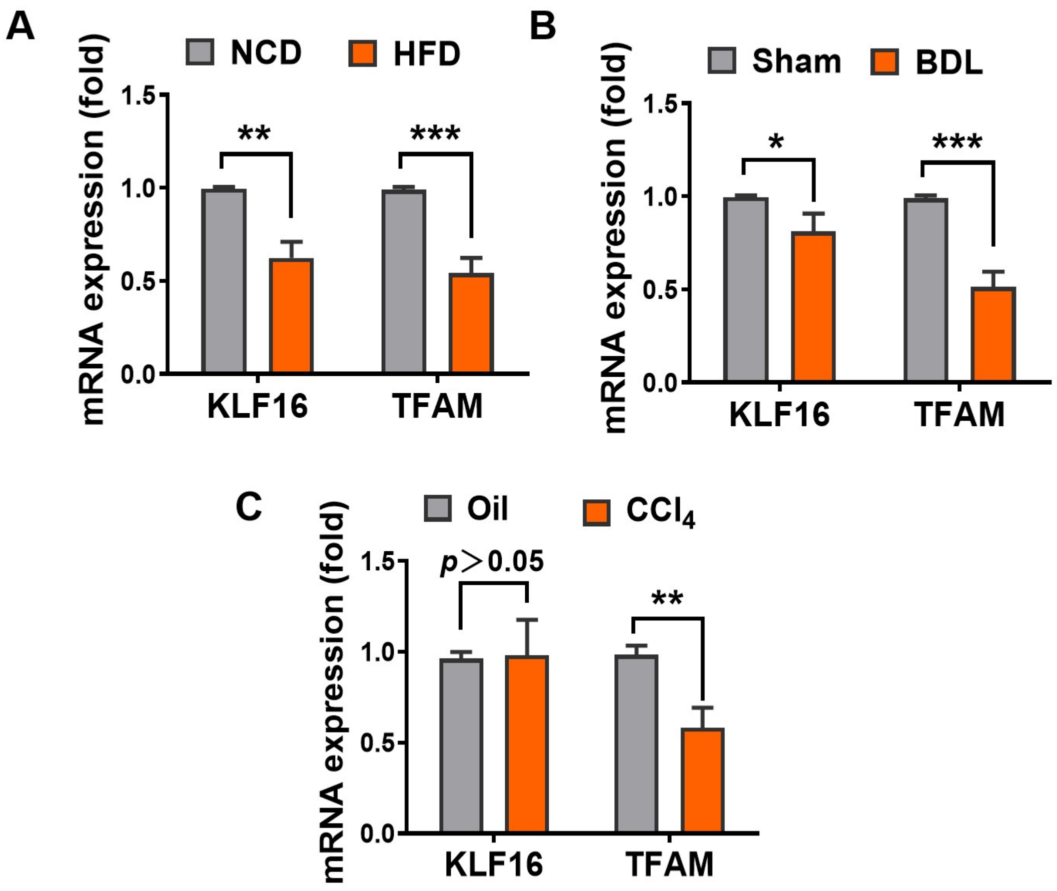

Figure S11. mRNA expression of KLF16 and TFAM in mice liver fibrosis models. qRT-PCR analysis of Nrf1 expression in macrophages isolated from three mice livers induced by HFD (A), CCl_4 injection (B) and BDL (C), N=6/group. Data were presented as the mean ± SD; *p < 0.05, **p < 0.01, ***p < 0.001.

Figure 1

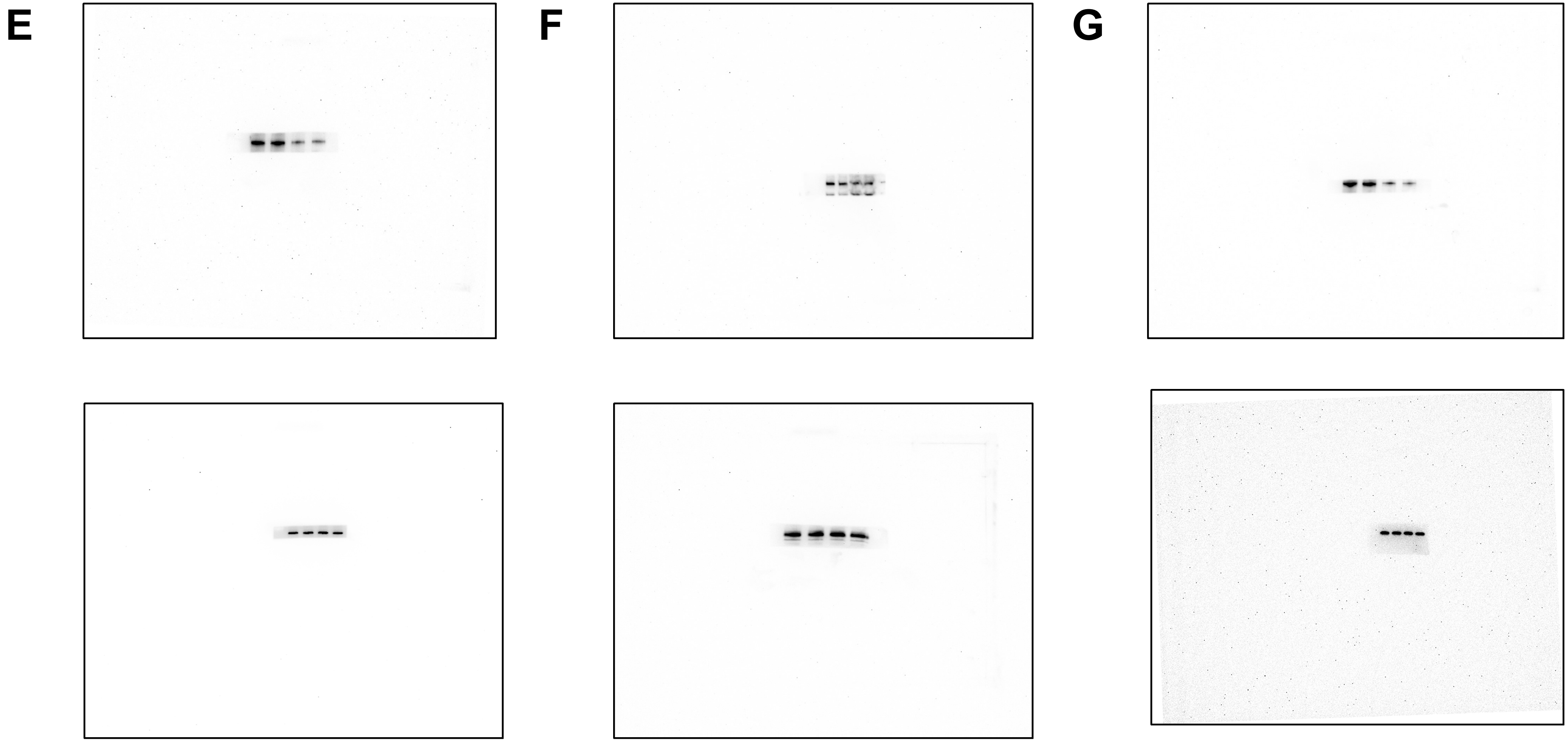

Figure 2A

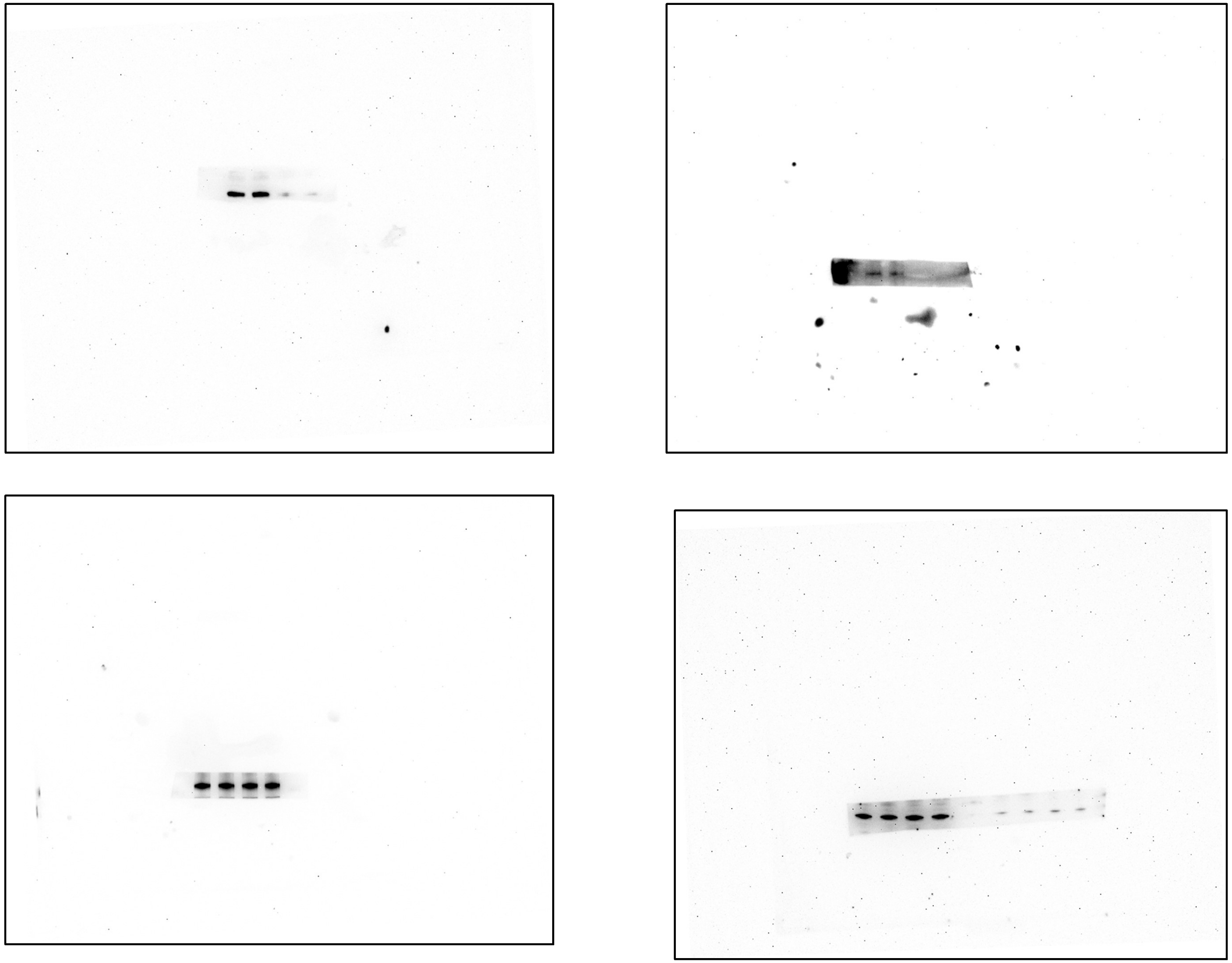

Figure 4

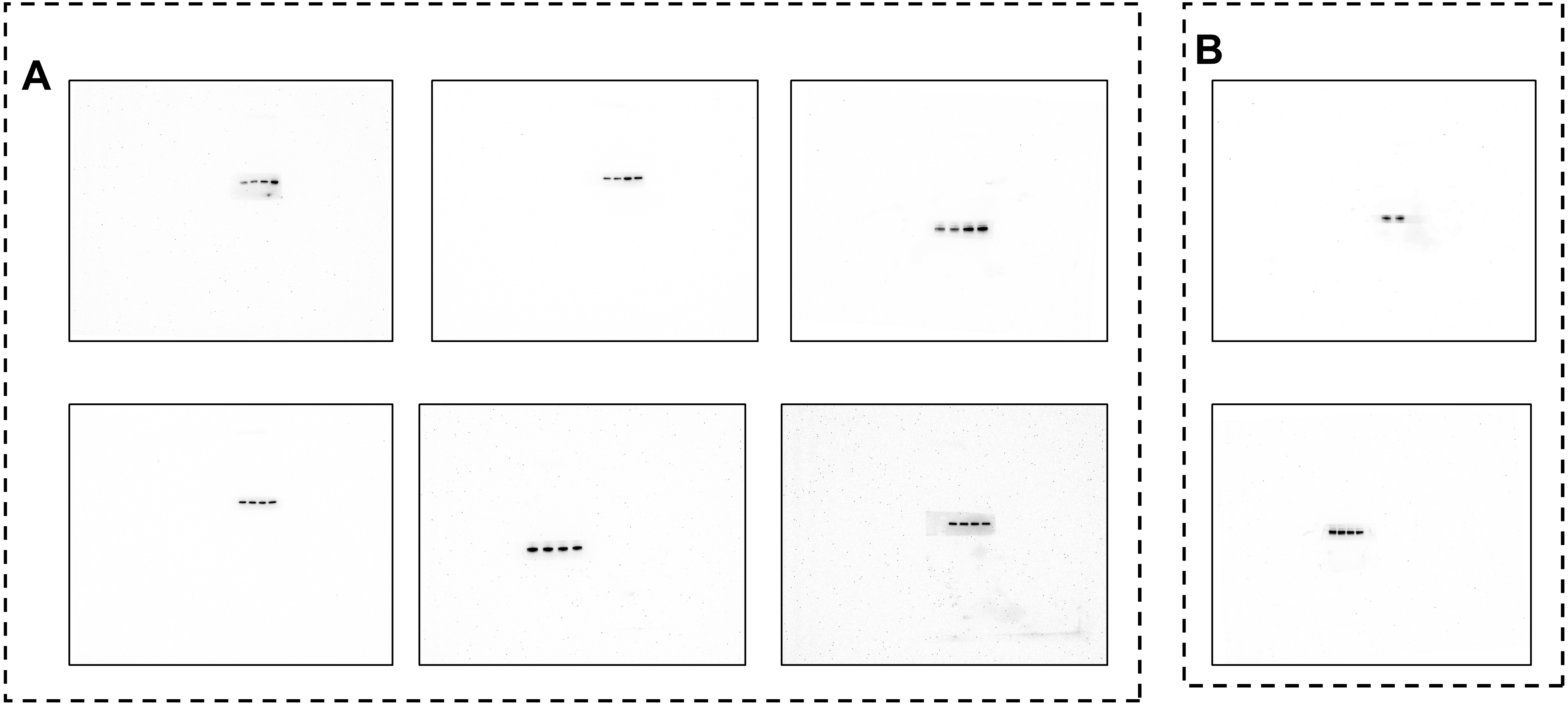

Figure 5

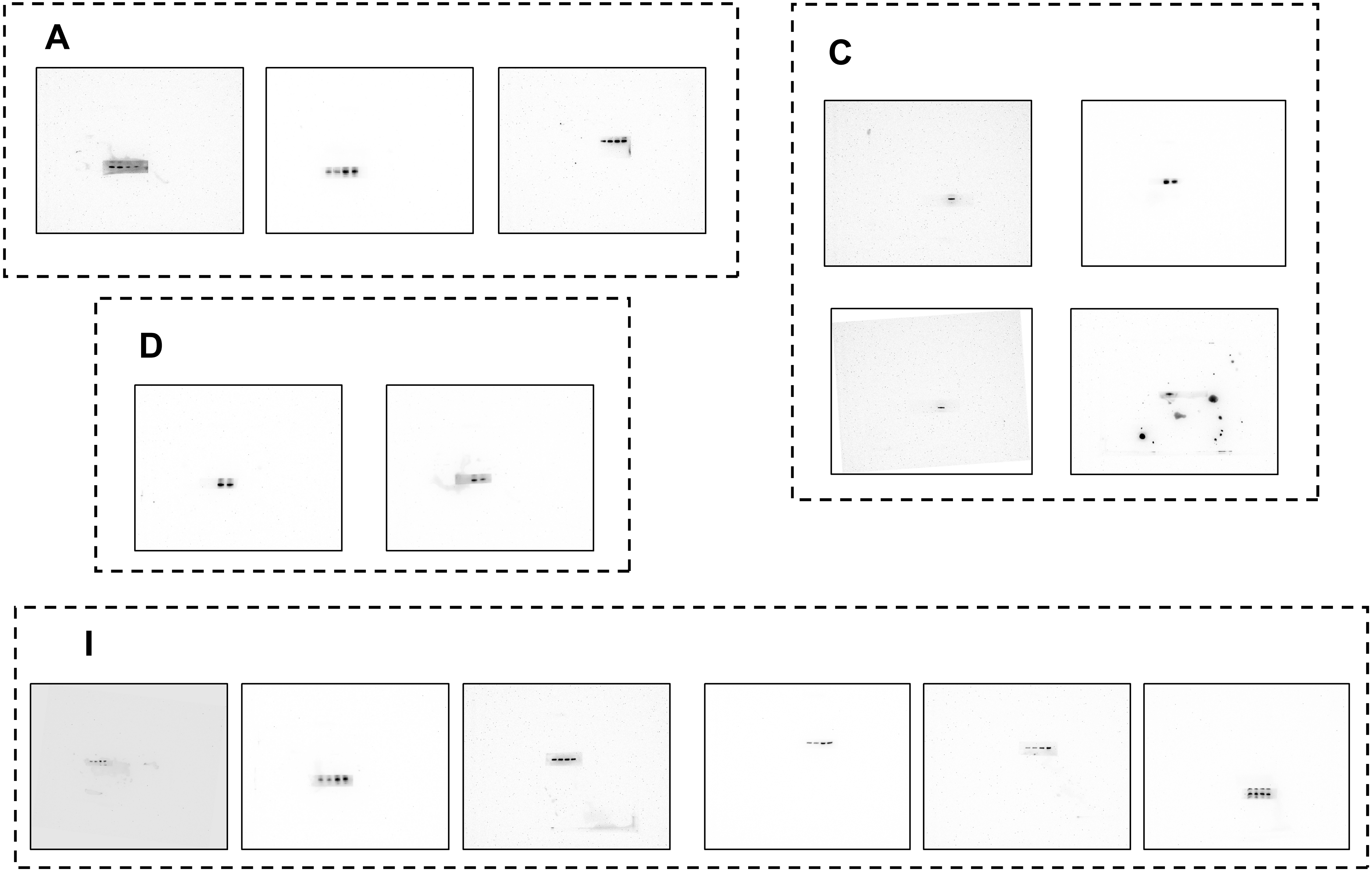

Figure 6

A

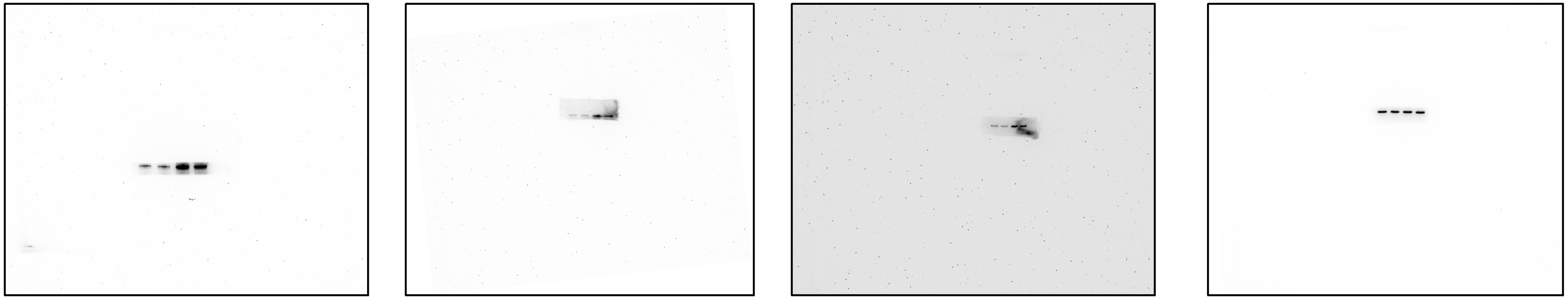

B

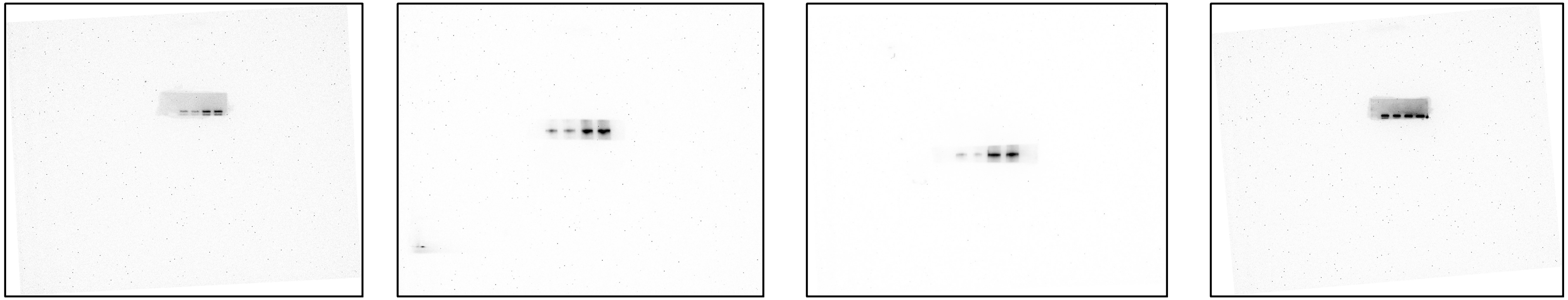

Figure 7

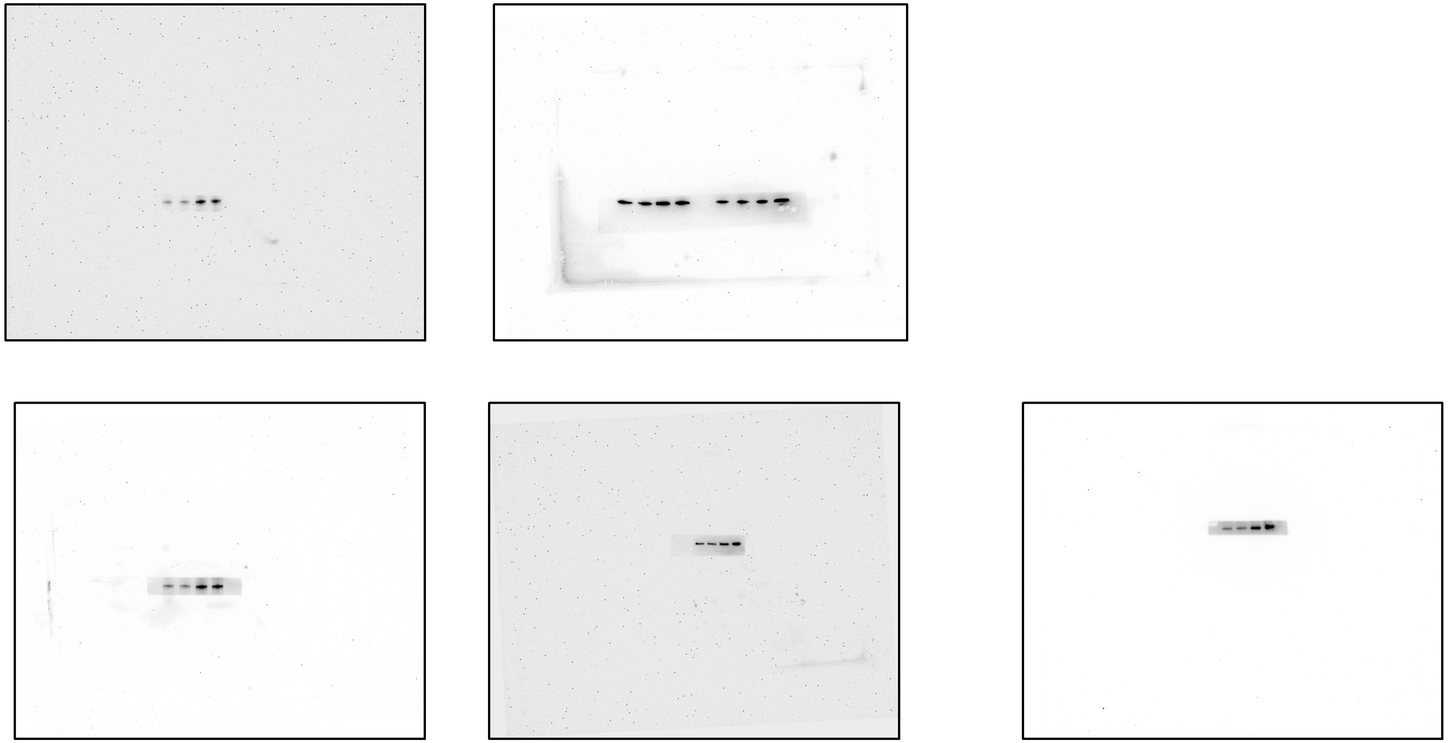

Supplement: Supplementary file 1 — Supplementary methods, figures and tables. [file thnov16p2269s1.pdf]
